# Autophagy Contributes to the Quality Control of Leaf Mitochondria

**DOI:** 10.1093/pcp/pcaa162

**Published:** 2020-12-23

**Authors:** Sakuya Nakamura, Shinya Hagihara, Kohei Otomo, Hiroyuki Ishida, Jun Hidema, Tomomi Nemoto, Masanori Izumi

**Affiliations:** 1 Center for Sustainable Resource Science (CSRS), RIKEN, Wako, 351-0198 Japan; 2 Exploratory Research Center on Life and Living Systems (ExCELLs), National Institute of Natural Sciences, Okazaki, 444-8787 Japan; 3 National Institute for Physiological Sciences, National Institutes of Natural Sciences, Okazaki, 444-8787 Japan; 4 Department of Physiological Sciences, The Graduate University for Advanced Study (SOKENDAI), Hayama, 240-0193 Japan; 5 Research Institute for Electronic Science, Hokkaido University, Sapporo, 001-0020 Japan; 6 Department of Applied Plant Science, Graduate School of Agricultural Sciences, Tohoku University, Sendai, 980-0845, Japan; 7 Department of Molecular and Chemical Life Sciences, Graduate School of Life Sciences, Tohoku University, Sendai, 980-8577, Japan; 8 PRESTO, Japan Science and Technology Agency, Kawaguchi, 322-0012 Japan

**Keywords:** Arabidopsis (*Arabidopsis thaliana*), Autophagy, Mitochondria, Mitophagy, Organelle quality control, Ultraviolet B

## Abstract

In autophagy, cytoplasmic components of eukaryotic cells are transported to lysosomes or the vacuole for degradation. Autophagy is involved in plant tolerance to the photooxidative stress caused by ultraviolet B (UVB) radiation, but its roles in plant adaptation to UVB damage have not been fully elucidated. Here, we characterized organellar behavior in UVB-damaged Arabidopsis (*Arabidopsis thaliana*) leaves and observed the occurrence of autophagic elimination of dysfunctional mitochondria, a process termed mitophagy. Notably, Arabidopsis plants blocked in autophagy displayed increased leaf chlorosis after a 1-h UVB exposure compared to wild-type plants. We visualized autophagosomes by labeling with a fluorescent protein-tagged autophagosome marker, AUTOPHAGY8 (ATG8), and found that a 1-h UVB treatment led to increased formation of autophagosomes and the active transport of mitochondria into the central vacuole. In *atg* mutant plants, the mitochondrial population increased in UVB-damaged leaves due to the cytoplasmic accumulation of fragmented, depolarized mitochondria. Furthermore, we observed that autophagy was involved in the removal of depolarized mitochondria when mitochondrial function was disrupted by mutation of the *FRIENDLY* gene, which is required for proper mitochondrial distribution. Therefore, autophagy of mitochondria functions in response to mitochondrion-specific dysfunction as well as UVB damage. Together, these results indicate that autophagy is centrally involved in mitochondrial quality control in Arabidopsis leaves.

## Introduction

Mitochondria produce much of the energy required by eukaryotic cells through respiration and serve other roles, such as regulating apoptotic cell death and acting as a reservoir for the second messenger calcium (Millar et al. 2008, Wang and Youle 2009, Ng et al. 2014). Given these functions, eukaryotes control the size, population, distribution and quality of mitochondria with regard to cell type, developmental stage and changing environmental conditions.

A number of evolutionarily conserved, mitochondrion-associated proteins optimize mitochondrial size, population and distribution in various species (Arimura 2018). In the budding yeast *Saccharomyces cerevisiae*, clustered mitochondria protein1 (Clu1p) is essential for maintaining proper mitochondrial distribution within the cell, as evidenced by the formation of abnormal clusters of mitochondria in *clu1Δ* deletion mutant cells (Fields et al. 1998). Clu1p homolog (CLUH) in mammals serves a similar function (Gao et al. 2014, Schatton et al. 2017), as does the Arabidopsis (*Arabidopsis thaliana*) homolog, named FRIENDLY, as clustered mitochondria also appear in *friendly* knock-out mutants (Logan et al. 2003). The *friendly* mutants show reduced shoot and root growth (El Zawily et al. 2014), highlighting the importance of this *CLU1* homolog in plant mitochondrial function and growth.

During mitochondrial respiration, the electron transport chain in the mitochondrial inner membrane produces membrane potential and ATP but is also a major site for production of reactive oxygen species (ROS), which can cause oxidative damage (Huang et al. 2016). Plant mitochondria further mediate an enzymatic reaction in photorespiration: the salvage cycle of photosynthetic biproducts, with the concomitant release of toxic ammonium ions (Eisenhut et al. 2019). The presence of these damaging compounds necessitates a quality control system that removes damaged plant mitochondria to prevent mitochondrial dysfunction and minimize the spread of toxic compounds. Although the quality control system for plant mitochondria remains poorly understood, an autophagy process termed mitophagy is well established in yeast and mammals, where it disposes of dysfunctional mitochondria.

Autophagy is an evolutionarily conserved process that removes cytoplasmic components for degradation in lytic organelles—lysosomes in animal cells and vacuoles in yeast and plant cells (Ohsumi 2001). This intracellular digestion process is important for recycling nutrients through bulk degradation of cytoplasmic components and for maintaining cellular homeostasis through the removal of non-performing organelles. In the major autophagy pathway termed macroautophagy, a double membrane-bound vesicle called an autophagosome encloses a portion of the cytoplasm. The outer membrane fuses to the lysosomal/vacuolar membrane and the inner-membrane structure called the autophagic body is digested.

The basic mechanism of autophagosome formation was originally described in budding yeast with the identification of *Autophagy* (*Atg*) genes (Klionsky et al. 2003). *Atg* genes that encode proteins required for the formation of autophagosomal membranes in budding yeast are designated ‘core’ *Atg* genes (*Atg1–Atg10*, *Atg12–14*, *Atg16* and *Atg18*; Nakatogawa et al. 2009). Most core *Atg* genes have orthologs widely distributed in the plant kingdom (Meijer et al. 2007). Studies of Arabidopsis *atg* mutants in core autophagy components show that they play similar functions as their yeast counterparts (Marshall and Vierstra 2018, Soto-Burgos et al. 2018, Yoshimoto and Ohsumi 2018).

The role of mitophagy has been extensively studied in yeast and mammals (Yamano et al. 2016, Pickles et al. 2018). In budding yeast, mitophagy is induced in the stationary phase of cell growth to optimize mitochondrial quality and quantity. As might be expected, disruption of mitophagy leads to increased ROS production and mutations in mitochondrial DNA (Kurihara et al. 2012). Mammalian autophagy is also essential for controlling mitochondrial quality, as its impairment is associated with several types of human neurodegenerative disorders (Pickles et al. 2018). The controlled removal of dysfunctional mitochondria via mitophagy is an important aspect of maintaining a healthy mitochondrial population to ensure cellular homeostasis. In plants, genetic evidence supporting the autophagy-dependent degradation of mitochondrial proteins and mitochondrial vesicles was reported in a study of accelerated leaf senescence caused by sugar starvation (Li et al. 2014). Various types of proteins and organelles are degraded during sugar starvation-induced senescence to facilitate nutrient recycling (Weaver and Amasino 2001, Wada et al. 2009, Law et al. 2018), but it remains uncertain whether some damaged plant mitochondria are selectively removed to maintain the quality of the entire mitochondrial population.

Plants are subjected to damage caused by sunlight, which contains strong visible light (wavelengths 400–700 nm) and ultraviolet B (UVB; wavelengths 280–315 nm) radiation. We reported previously that the autophagic degradation of chloroplasts, termed chlorophagy, eliminates collapsed chloroplasts after damage by UVB or visible light (Izumi et al. 2017, Nakamura et al. 2018). Here, we directly compared the effects induced by UVB and strong visible light on wild-type (WT) and *atg* mutants; we determined that *atg* mutants affecting the core autophagy machinery are more sensitive to UVB damage at levels of UVB radiation that do not normally induce active chlorophagy in WT. These results indicated that the lack of chlorophagy for photodamaged chloroplasts in *atg* mutants does not fully explain their UVB-sensitive phenotype. Therefore, we hypothesized that plant autophagy likely involves the turnover of other organelles in response to UVB damage and tested this hypothesis. Our observations of fluorescently labeled organelles revealed an increase in the mitochondrial population in UVB-damaged *atg* mutant leaves. Detailed microscopy of mitochondria, autophagy markers and a mitochondrial probe that stains functional mitochondria revealed that UVB damage induced the active transport of mitochondria into vacuole in WT, whereas fragmented and depolarized mitochondria accumulated in the cytoplasm in *atg* mutant leaves. Furthermore, the mitochondrion-specific defects caused by a mutation of *FRIENDLY* were enhanced upon loss of autophagy, supporting the notion that autophagy alleviates mitochondrial dysfunction caused by the *friendly* mutation. Our results establish that autophagy participates in mitochondrial quality control in Arabidopsis.

## Results

### The loss of chlorophagy does not fully explain the hypersensitivity of core *atg* mutants to UVB-induced damage

To better understand the roles of autophagy in plant responses to photooxidative stress, we began by comparing the phenotypes caused by exposure to UVB and strong visible light in multiple Arabidopsis lines carrying mutations of core ATG components (Fig.�1A). We subjected WT plants and the *ATG* T-DNA insertion mutants *atg2*, *atg5* and *atg7* to UVB exposure (1.5 W m^−2^) for 1 or 2 h and allowed them to recover for 7 d before taking photographs. We noticed that *atg* plants showed worse leaf chlorosis compared to WT plants (Fig.�1A). By contrast, *atg* mutant plants did not show such sensitivity to treatment with high visible light (HL) consisting of 2,000 �mol m^−2^ s^−1^ for 2 h (Fig.�1A, bottom).

**Fig. 1:**
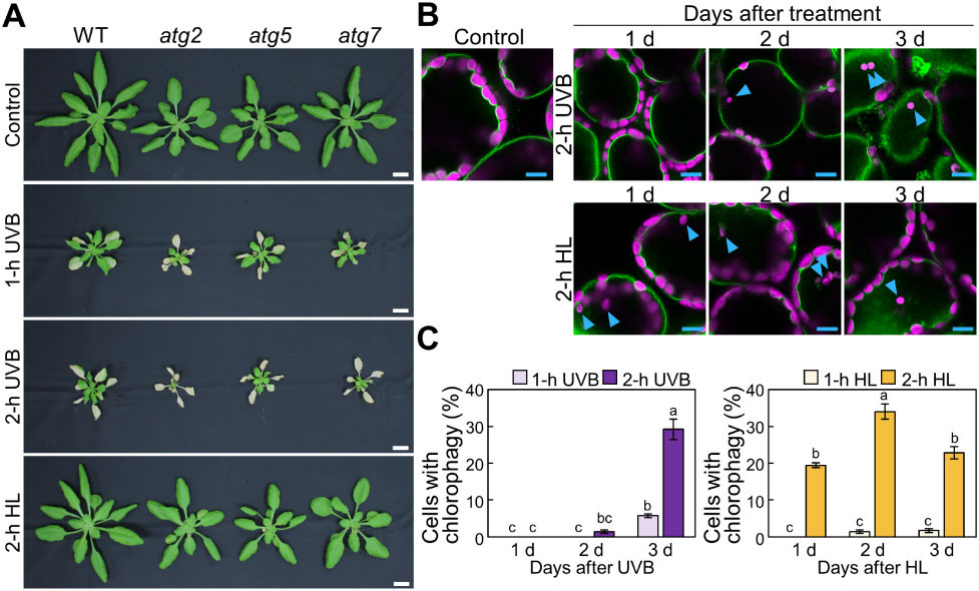
The loss of chlorophagy is not sufficient to explain the UVB-sensitive phenotype of Arabidopsis mutants harboring mutations of core autophagy genes. (A) Visual phenotypes of Arabidopsis plants 7 d before (control), or after 1-h UVB, 2-h UVB or 2-h HL exposure. WT, *atg2*, *atg5* and *atg7* plants were exposed to UVB (wavelength 280–315 nm) of 1.5 W m^−2^ for 1 or 2 h, or to high visible light consisting of 2,000 �mol m^−2^ s^−1^. Scale bars = 10 mm. (B) Confocal images of mesophyll cells expressing the tonoplast marker *VHP1-mGFP* from either nontreated control leaves or leaves 1, 2 or 3 d after UVB treatment (1.5 W m^−2^) or HL (2,000 μmol m^−2^ s^−1^) treatment for 2 h. Blue arrowheads indicate vacuole-enclosed chloroplasts. Green, VHP1-mGFP; magenta, chlorophyll autofluorescence. Only merged images are shown. Scale bars = 10 �m. (C) Proportion of cells with vacuole-enclosed chloroplasts in a fixed region, obtained from images described in (B) (� SE, *n* = 4). Different letters in each graph denote significant differences based on Tukey’s test (*P* < 0.05).

We previously demonstrated that HL and UVB damage induce the elimination of damaged chloroplasts via chlorophagy, which requires ATG5 or ATG7 function (Nakamura and Izumi 2018). Therefore, we next determined the extent of chlorophagy in each condition. As we previously showed that the frequency of vacuolar membrane-enclosed chloroplasts reflects the activity of chlorophagy, we monitored chlorophagy in transgenic plants expressing the vacuolar membrane marker VACUOLAR H^+^-PYROPHOSPHATASE 1 (VHP1) fused to monomeric green fluorescent protein (mGFP; Fig.1B arrowheads; Segami et al. 2014, Nakamura et al. 2018, Kikuchi et al. 2020). Using this marker, we observed vacuole-enclosed chloroplasts, showing that chlorophagy is induced earlier in WT plants exposed to 2 h HL compared to 2 h UVB; indeed, many vacuole-enclosed chloroplasts appeared after 1 d of HL treatment or after 3 d following UVB exposure (Fig.�1C). These results therefore indicated that chlorophagy is more active in HL-damaged leaves than in UVB-damaged leaves, which does not easily align with the more sensitive phenotypes seen in *atg2*, *atg5* or *atg7* mutants in response to UVB relative to HL treatment (Fig.�1A). The distinct phenotypes induced by UVB and HL further suggested that the loss of chlorophagy in *atg* mutant plants does not fully explain their susceptibility to UVB damage. We therefore hypothesized that autophagy may degrade other cellular components damaged by UVB.

To confirm the reduced tolerance of mutants defective in autophagy to UVB damage, we determined the phenotypes of the additional *atg* mutants *atg10*, *atg12a*, *atg12b* and *atg18a* (Supplementary Fig. S1A, B). Compared to WT plants, cell death was elevated in the leaves of all *atg* mutants tested after UVB treatment (Supplementary Fig. S1A). We measured the maximum quantum yields of photosystem II (PSII) (*F*_v_/*F*_m_) since previous studies showed a decline in *F*_v_/*F*_m_ in response to the accelerated leaf chlorosis symptoms in *atg* mutant leaves (Izumi et al. 2017, Hirota et al. 2018). Consistent with the visible phenotypes, the *F*_v_/*F*_m_ ratio declined in *atg* mutant leaves compared to WT leaves 7 d after a 1- or 2-h exposure to UVB (Supplementary Fig. S1B). The *photolyase 1* (*phr*) mutant, also named *UV resistance 2* (*uvr2*), which lacks the cyclobutene pyrimidine dimer photolyase activity necessary to repair UVB-induced DNA damage, was used as a well-established UVB-sensitive mutant line (Supplementary Fig. S1C, D; Landry et al. 1997, Willing et al. 2016). The *phr* and *atg* mutants all showed similar UVB sensitivity (Supplementary Fig. S1A, B). These results support the importance of autophagy in the plant response to UVB damage.

### UVB damage causes the accumulation of mitochondria in *atg* mutants

The comparison of plant phenotypes and chlorophagy activity after UVB and HL damage (Fig.�1) strongly suggested that autophagy is involved in the quality control of multiple cellular components in addition to chloroplasts when plants are exposed to UVB. To investigate this possibility, we observed nuclei, plastid nucleoids, peroxisomes and mitochondria labeled with green fluorescent protein (GFP) 1 d after a 1-h exposure to UVB (1.5 W m^−2^), i.e. at a time when chlorophagy has not yet occurred in WT plants (Fig.�1C). We expected that the behavior of organellar populations or organelle sizes might be different between WT and *atg5* plants if autophagy is the main degradation route for damaged organelles in response to UVB.

To visualize nuclei, we used a previously generated line expressing a nucleus-localized marker: the N-terminal GFP-tagged At4g19150 protein (an ankyrin-like protein; *Pro35S:GFP-At4g19150*; Cutler et al. 2000). The resulting nucleus-targeted GFP fluorescence showed no apparent differences between untreated and UVB-exposed leaves in either WT or *atg5* plants (Fig.�2A). Although the average nuclear volume calculated from the nucleus-targeted GFP signals decreased slightly in WT and increased in *atg5* plants 1 d after the UVB exposure, we did not detect a statistically significant difference between samples (Fig.�2B). We next turned to plastid nucleoids, which we visualized in transgenic plants expressing a fusion between SWI/SNF complex B protein 2 (SWIB2, a plastid nucleoid marker; Melonek et al. 2012) and GFP (*Pro35S:SWIB2-GFP*; Fig.�2C), since previous studies revealed that autophagy can degrade a portion of a chloroplast without chlorophagic dismantling of the entire chloroplast (Ishida et al. 2008, Wang et al. 2013, Michaeli et al. 2014). An evaluation of the number of plastid nucleoids from three-dimensional images showed no differences between WT and *atg5* plants in either untreated control leaves or leaves exposed to UVB (Fig.�2D), suggesting that partial autophagy of chloroplast components does not play a role in the degradation of plastid nucleoids in UVB-damaged leaves.

**Fig. 2:**
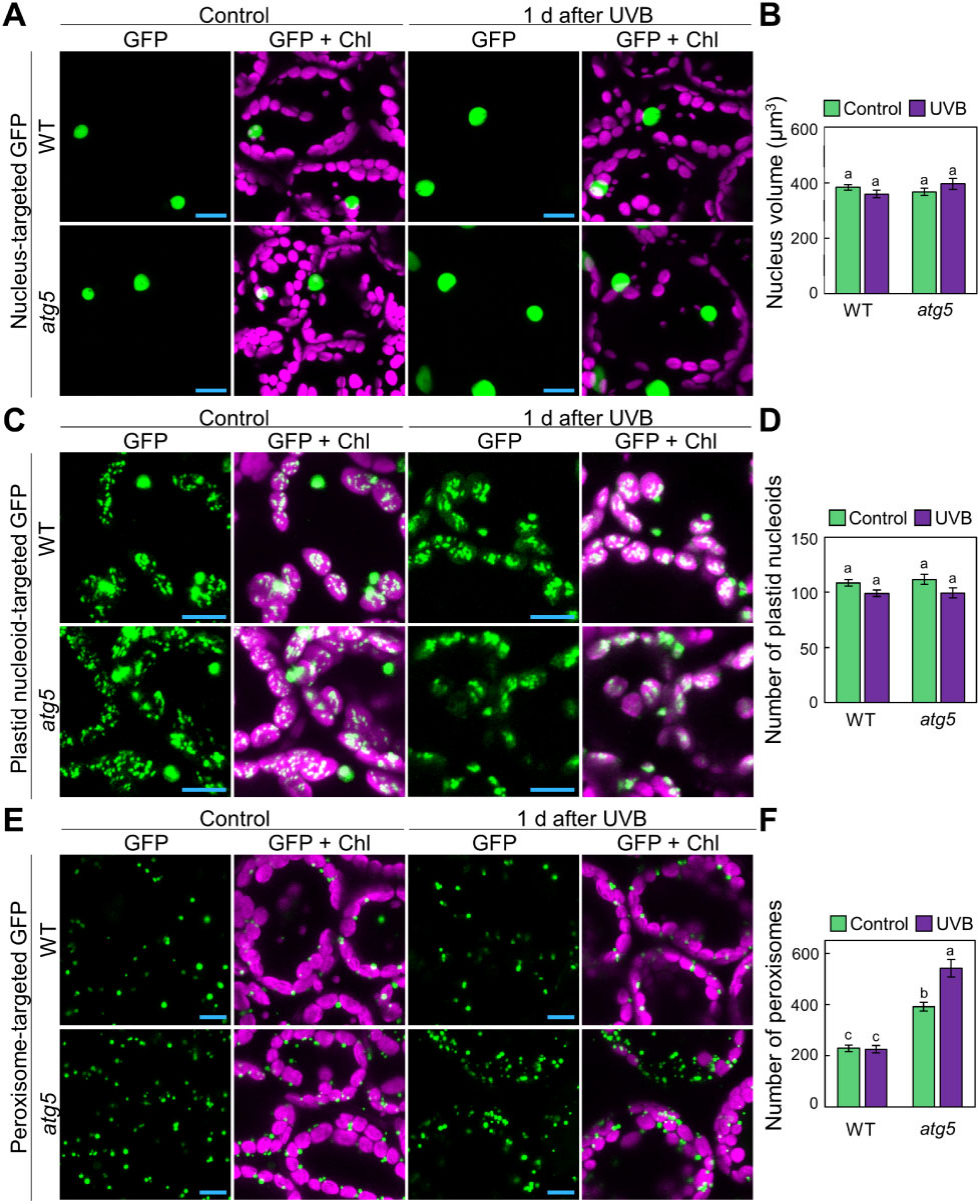
Behavior of plastid nucleoids, nuclei and peroxisomes in UVB-damaged leaves. (A) Confocal images of mesophyll cells expressing nucleus-targeted GFP from WT and *atg5* untreated control plants or plants 1 d after a 1-h UVB (1.5 W m^−2^) exposure. (B) Mean nucleus volumes obtained from the three-dimensional images described in (A) (� SE, *n* = 4). (C) Confocal images of mesophyll cells expressing plastid nucleoid-targeted GFP from WT or *atg5* untreated control plants or plants 1 d after a 1-h UVB (1.5 W m^−2^) exposure. (D) Number of plastid nucleoids, obtained from the three-dimensional images described in (C) (� SE, *n* = 4). (E) Confocal images of mesophyll cells expressing peroxisome-targeted GFP from WT and *atg5* untreated control plants or plants 1 d after a 1-h UVB (1.5 W m^−2^) exposure. (F) Number of peroxisomes obtained from the three-dimensional images described in (E) (� SE, *n* = 4). For confocal images, orthogonal projections created from *z*-stack images are shown. Green, GFP; magenta, chlorophyll autofluorescence (Chl). Scale bars = 10 �m. Different letters in each graph denote significant differences based on Tukey’s test (*P* < 0.05).

However, we detected a clear difference in the number of peroxisomes labeled by GFP-MULTIFUNCTIONAL PROTEIN 2 (MFP2; Cutler et al. 2000) fusion between WT and *atg5* plants. Peroxisome number was higher in *atg5* leaves than in WT leaves in the absence of UVB treatment (Fig.�2E, F), which is consistent with previous studies that reported the participation of autophagy in quality control of leaf peroxisomes (Kim et al. 2013, Shibata et al. 2013, Yoshimoto et al. 2014). After UVB damage, the accumulation of peroxisomes further increased in *atg5* leaves, indicating the activation of the mechanism of peroxisome-targeting autophagy termed pexophagy in response to UVB damage.

Notably, UVB exposure also affected the behavior of leaf mitochondria (Fig.�3). We subjected WT and *atg5* plants expressing a construct consisting of the mitochondrial matrix marker ISOCITRATE DEHYDROGENASE (IDH) fused to GFP (*ProIDH:IDH-GFP*; Hirota et al. 2018), to a 1-h UVB treatment. We observed a larger mitochondrial population in *atg5 ProIDH:IDH-GFP* leaves than in WT leaves 1 d after treatment (Fig.�3A). We then counted the numbers of mitochondria in a given volume from three-dimensional images (Fig.�3B). In WT plants, mitochondrial GFP spots decreased by 85.3 � 6.4% 1 d after UVB exposure. By contrast, in *atg5* plants, the number of mitochondria increased by 159.0 � 14.0% after UVB damage (Fig.�3B). Unlike peroxisomes, the number of leaf mitochondria was similar across all genotypes in untreated conditions (Fig.�3A, B).

**Fig. 3:**
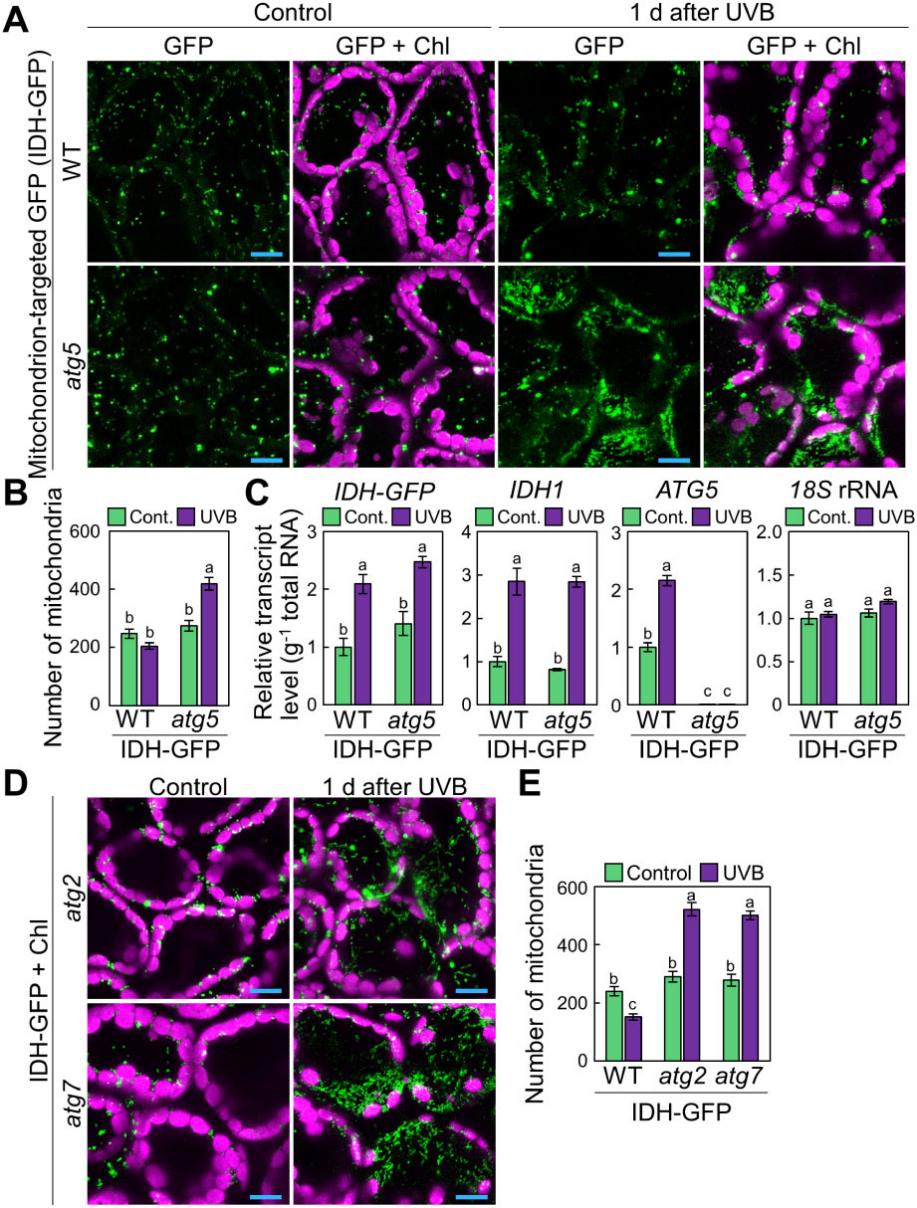
Autophagy deficiency causes an increase in mitochondrial population in UVB-damaged leaves. (A) Confocal images of mesophyll cells expressing mitochondrion-targeted isocitrate dehydrogenase-GFP (IDH-GFP) from WT and *atg5* untreated control plants or plants 1 d after a 1-h UVB (1.5 W m^−2^) exposure. (B) Mean numbers of mitochondria obtained from the three-dimensional images described in (A) (� SE, *n* = 4). (C) Transcript levels for *IDH-GFP*, *IDH1* and *ATG5* in leaves of WT and *atg5* untreated control plants or plants 1 d after a 1-h UVB exposure (� SE, *n* = 3). Transcript levels of the respective genes are shown relative to the values from WT control leaves, which are set to 1. The level of *18S* rRNA was measured as an internal control. (D) Confocal images of mesophyll cells expressing *IDH-GFP* from *atg2* and *atg7* untreated control plants or plants 1 d after a 1-h UVB exposure. (E) Number of mitochondria obtained from the three-dimensional images described in (D) (� SE, *n* = 4). For confocal images, orthogonal projections created from *z*-stack images are shown. Green, GFP; magenta, chlorophyll autofluorescence (Chl). Scale bars = 10 �m in each image. Different letters in each graph denote significant differences based on Tukey’s test (*P* < 0.05).

We next focused on the changes in mitochondrial populations caused by UVB damage since the roles of plant autophagy in mitochondrial quality control have not been well characterized. We confirmed that the transcript levels of the *IDH-GFP* transgene and *IDH1* were similar between genotypes in each condition (Fig.�3C). Thus, the differences in the number of fluorescently labeled mitochondria between WT and *atg5* plants in response to UVB damage cannot be attributed to differences in *IDH-GFP* transcript levels. The increase in the size of the mitochondrial population seen in UVB-damaged *atg5* leaves also occurred in the two other *atg* mutants, *atg2* and *atg7* (increase by 189.0 � 9.0% and 188.5 � 16.3% compared to untreated control leaves, respectively), indicating a general defect due to the loss of autophagy components (Fig.�3D, E).

To assess whether the fluorescent mitochondrial marker proteins used contributed in any way to the observed differences between genotypes, we tested an additional GFP fusion targeted to mitochondria by adding the mitochondrion-targeting signal peptide of yeast cytochrome oxidase subunit IV (cox4) to GFP and expressing the fusion cassette under the control of the *Cauliflower mosaic virus* (CaMV) *35S* promoter (*Pro35S:MT-GFP*; Kohler et al. 1997; Supplementary Fig. S2). Consistent with the results obtained with *ProIDH:IDH-GFP* transgenic plants (Fig.�3A), we observed an increase in mitochondrial number in the leaves of UVB-exposed *atg5* and *atg7* plants expressing *Pro35S:MT-GFP* (Supplementary Fig. S2).

### Fragmented mitochondria accumulate in UVB-exposed *atg* mutants

In mammals, the shape of mitochondria is an important factor in controlling their removal, as dysfunctional mitochondria become fragmented, and such mitochondrial fragments are then easily engulfed by autophagosomes and degraded (Twig et al. 2008, Tanaka et al. 2010, Kageyama et al. 2012). To determine whether changes to mitochondrial shape occurred after UVB damage, we used an unconventional type of two-photon excitation microscope equipped with a confocal spinning disk unit (Otomo et al. 2015). Two-photon excitation is advantageous to observe deep areas of living tissues (Denk et al. 1990), while multipoint scanning with a confocal spinning-disk unit allows high-speed scanning and a superior axial (spatial) resolution compared to conventional, single-point scanning with a mirror galvanometer (Maddox et al. 2003). Thus, two-photon excitation confocal microscopy with a spinning-disk unit facilitates the precise observation of the three-dimensional structure of mitochondria in living Arabidopsis cells.

Using this system, we obtained high-quality three-dimensional images of mitochondria from plants expressing the *Pro35S:MT-GFP* transgene (Fig.�4A). We confirmed that the number of mitochondria was elevated in UVB-damaged leaves of *atg5* and *atg7* plants relative to WT plants (Fig.�4B). We then compared the distribution of mitochondrial sizes between the different conditions and noted that the proportion of mitochondria smaller than 2.0 �m^3^ increased after UVB damage in *atg* but not in WT leaves (Fig.�4C). These results confirmed the cytoplasmic accumulation of small, fragmented mitochondria in UVB-damaged *atg* plants.

**Fig. 4:**
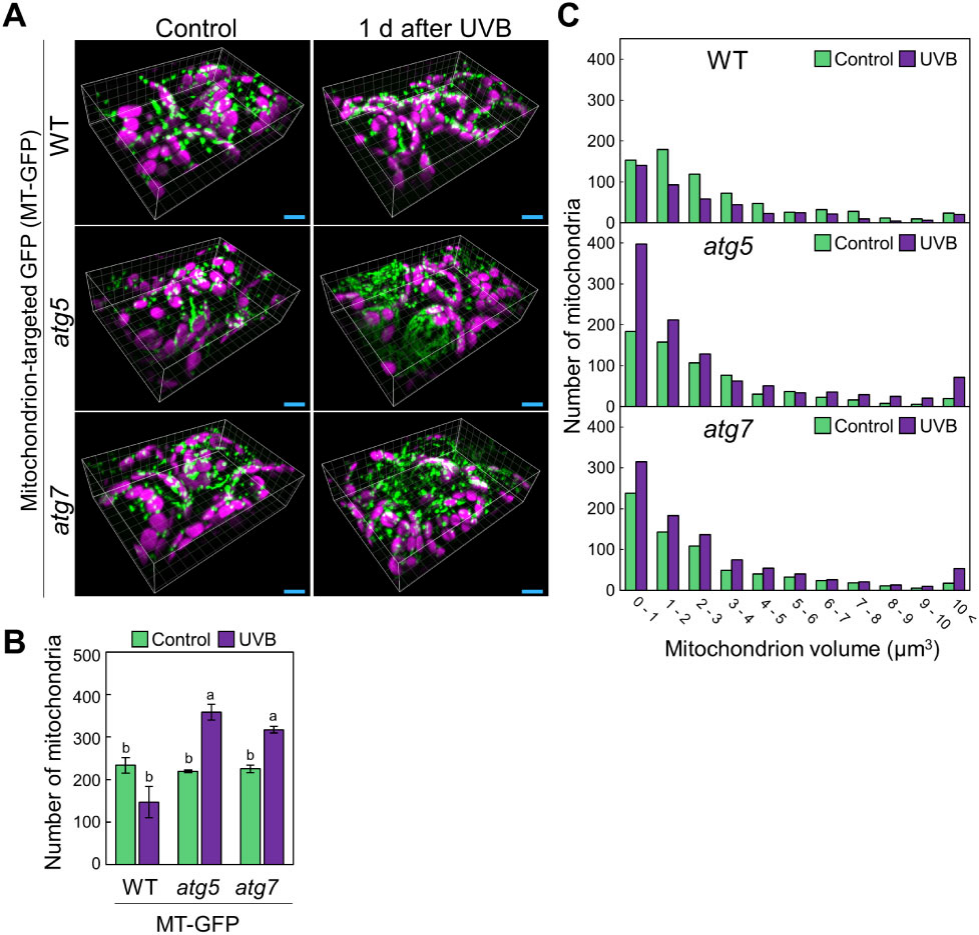
Autophagy deficiency causes an increase in the fraction of small mitochondria in UVB-damaged leaves. (A) Three-dimensional images of mesophyll cells expressing mitochondrial matrix-targeted GFP (*MT-GFP*) from WT, *atg5* and *atg7* untreated control plants or plants 1 d after a 1-h UVB exposure (1.5 W m^−2^) obtained using a two-photon excitation confocal microscope equipped with a spinning-disc unit. Green, GFP; magenta, chlorophyll autofluorescence (Chl). Scale bars = 10 �m. (B) Mean number of mitochondria from observations described in (A) (� SE, *n* = 3). Different letters denote significant differences based on Tukey’s test (*P* < 0.05). (C) Histograms showing the distribution of mitochondrial volumes obtained from the three-dimensional images in (A).

To corroborate the results of our fluorescent protein-based observations of mitochondria via confocal microscopy, we examined mitochondria in fixed leaves by transmission electron microscopy (TEM; Fig.�5A). We observed many mitochondria accumulating in the cytoplasm of *atg5* mesophyll cells 1 d after 1-h UVB exposure, relative to WT leaves (Fig.�5A, arrowheads). When we measured the sizes of mitochondria in these TEM images (Fig.�5B), the fraction of mitochondria smaller than 0.6 �m^2^ increased after UVB damage in the leaves of *atg5*, but not of WT plants, consistent with the results of the fluorescent protein marker-based observation (Figs.�3, 4).

**Fig. 5:**
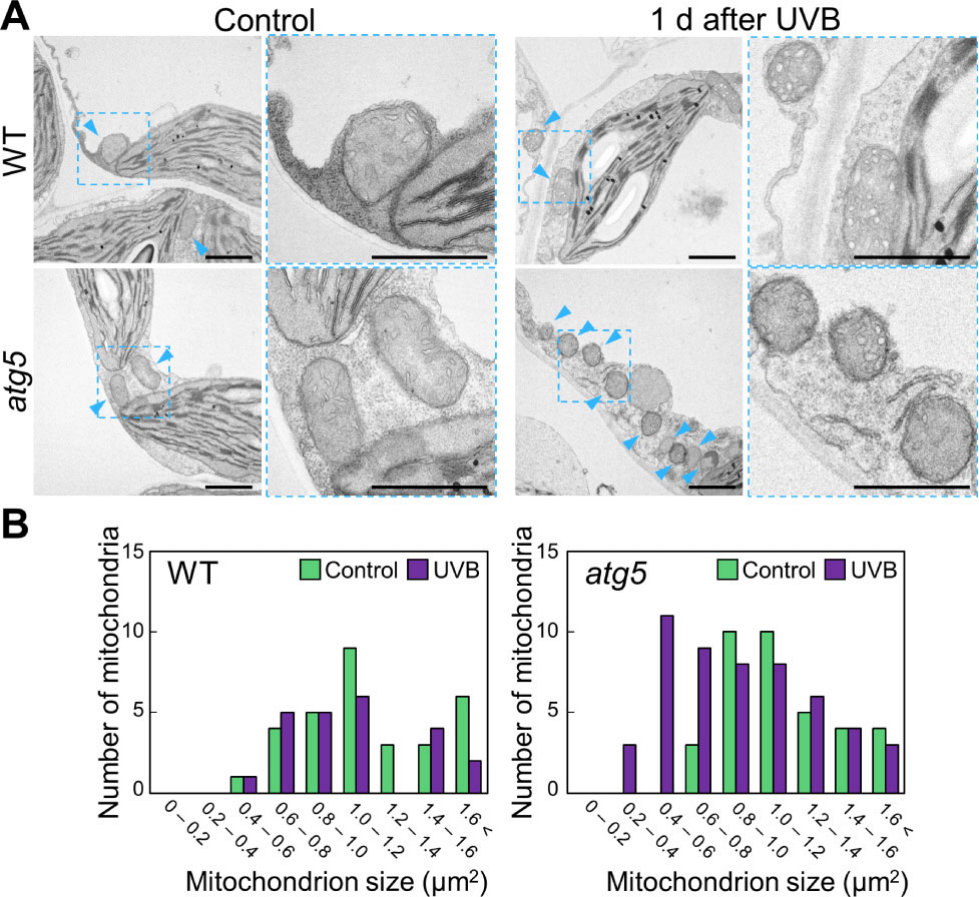
Electron microscopy of the cytoplasmic accumulation of small mitochondria in UVB-damaged *atg5* leaves. (A) Transmission electron micrographs from mesophyll cells of WT and *atg5* untreated control plants or plants 1 d after UVB exposure (1.5 W m^−2^) for 1 h. Blue arrowheads indicate mitochondria in cytoplasm. Scale bars = 2 �m. The area indicated by a dashed blue box is expanded to the right of each panel. (B) Histograms showing the distribution of mitochondrial sizes obtained from the TEM images in (A).

We also checked whether mitochondrial behavior was affected by HL damage (Supplementary Fig. S3). Mitochondrial number did not increase 1 d after HL exposure for 2 h (Supplementary Fig. S3), although this treatment actively induced the vacuolar accumulation of chloroplasts via autophagy (Fig.�1B, C). Therefore, relative to chloroplasts, mitochondria are likely more sensitive to UVB damage and less sensitive to strong visible light.

### UVB damage increases the association of autophagic membranes with mitochondria

The ATG8 ubiquitin-like protein family is an integral component of autophagosomal membranes (Ohsumi 2001). The Arabidopsis genome encodes nine ATG8 members (named *ATG8a–ATG8i*). With the exception of *ATG8d*, all Arabidopsis *ATG8* genes exhibited elevated transcript levels in UVB-damaged leaves (Supplementary Fig. S4A). In leaves expressing *GFP-ATG8a* under the constitutive *UBIQUITIN10* promoter (*ProUBQ10:GFP-ATG8a*), cytoplasmic autophagy structures increased in response to UVB damage, as evidenced by GFP-ATG8a puncta (Supplementary Fig. S4B), indicating that autophagosome formation is activated after UVB damage.

To directly evaluate the occurrence of autophagosome-mediated transport of mitochondria, we generated transgenic plants expressing *IDH-GFP* driven by the *IDH* promoter (*ProIDH:IDH-GFP*) along with a construct fusing *Red Fluorescent Protein* (*RFP*) and *ATG8a*, placed under the control of the *UBQ10* promoter (*ProUBQ10:RFP-ATG8a*). Consistent with the results in *GFP-ATG8a*-expressing plants (Supplementary Fig. S4B), RFP-ATG8a-labeled puncta were more numerous in the leaves of UVB-damaged plants than in those of untreated control plants, in which some RFP-ATG8a spots were associated with mitochondria (Fig.�6A, arrowheads). We quantified the number of RFP-ATG8a puncta and mitochondrion-associated RFP-ATG8a puncta in the cytoplasm (Fig.�6B). This evaluation confirmed the activation of autophagosome formation and their increased association with mitochondria in response to UVB exposure.

**Fig. 6:**
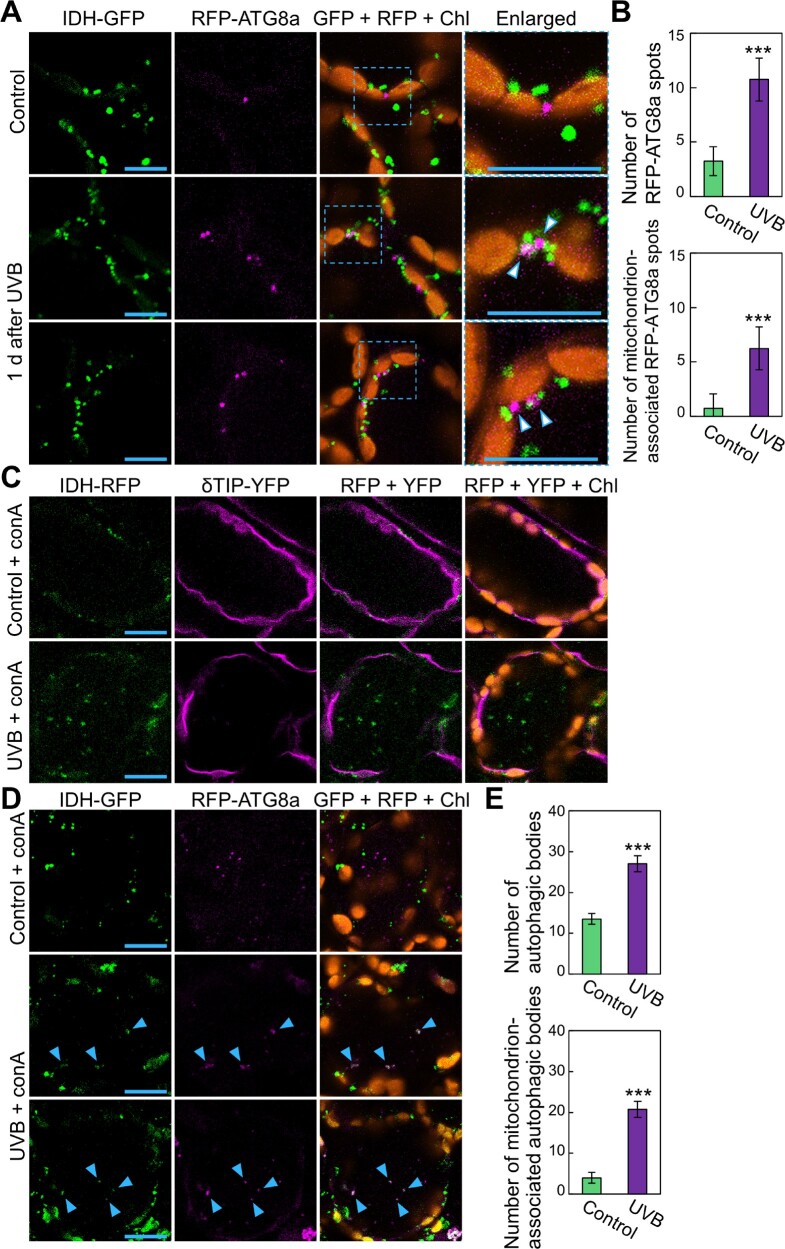
UVB damage activates autophagosome-mediated transport of mitochondria to the central vacuole. (A) Confocal images of mesophyll cells expressing mitochondrial *IDH-GFP* and autophagosomal RFP-ATG8a from untreated control plants or plants 1 d after a 1-h UVB (1.5 W m^−2^) exposure. Green, IDH-GFP; magenta, RFP-ATG8a; orange, chlorophyll autofluorescence (Chl). For UVB treatment, two representative images are shown. (B) Number of autophagic structures (top) and mitochondrion-associated autophagic structures (bottom) from (A) (� SE, *n* = 4). (C) Confocal images of mesophyll cells expressing mitochondrial *IDH-RFP* and the tonoplast membrane marker *δTIP-YFP* from ConA-treated leaves. Leaves of untreated control plants or plants immediately after a 1-h UVB exposure were subjected to a 1-d incubation with ConA. Green, IDH-RFP; magenta, δTIP-YFP; orange, chlorophyll autofluorescence. (D) Confocal images of mesophyll cells expressing mitochondrial *IDH-GFP* and autophagosomal *RFP-ATG8a* from ConA-treated leaves. Leaves of untreated control plants or plants immediately after a 1-h UVB (1.5 W m^−2^) exposure were subjected to a 2-d incubation with ConA. Green, IDH-GFP; magenta, RFP-ATG8a; orange, chlorophyll autofluorescence (Chl). For UVB treatment, two representative images are shown. Blue arrowheads indicate mitochondria colocalized with autophagosomal RFP-ATG8a signals in the vacuole. (E) Number of autophagic bodies (top) and mitochondrion-associated autophagic bodies (bottom) from (D) (� SE, *n* = 4). Throughout, scale bars = 10 μm; asterisks denote significant differences between control and UVB-treated plants based on Student’s *t*-test (***P < 0.001). The area indicated by a dashed blue box is expanded to the right of each panel.

Concanamycin A (ConA) is an inhibitor of vacuolar H^+^-ATPase activity that allows the stabilization of autophagic bodies labeled by fluorescent protein-ATG8 fusions in the vacuole (Ishida et al. 2008). To determine whether mitochondria are transported into the vacuole via autophagosomes after UVB damage, we therefore incubated leaves from UVB-exposed plants with a ConA solution. First, we used plants expressing the mitochondrial *IDH-RFP* construct along with the tonoplast marker *Yellow Fluorescent Protein* (*YFP*)-*DELTA TONOPLAST INTRINSIC PROTEIN* (δTIP; *ProδTIP:δTIP-YFP*; Nakamura et al. 2018). When we exposed these plants to UVB and then incubated their leaves with ConA, the mitochondrial RFP signals that appeared to move randomly were detected inside the vacuole (Fig.�6C), indicating that the mitochondria were transported into the vacuolar lumen after UVB damage.

We then subjected plants expressing mitochondrial *IDH-GFP* and autophagosomal *RFP-ATG8a* to ConA treatment. In control leaves, ConA treatment revealed the accumulation of autophagic bodies labeled by RFP-ATG8a in the central area of mesophyll cells (Fig.�6D). In UVB-damaged leaves, we observed co-localization of mitochondrial IDH-GFP signals with autophagosomal RFP-ATG8a signals in the vacuole (Fig.�6D), indicating that mitochondria were incorporated into the vacuolar lumen as the cargo of autophagic bodies. Quantification of the number of autophagic bodies showed that mitochondrion-associated autophagic bodies in the vacuole increased in response to UVB damage (Fig.�6E).

GFP and RFP differ in their sensitivity to lytic activity, with RFP being more tolerant of both lytic activity and low pH environments (Kimura et al. 2007). We therefore assessed the movement of mitochondria in transgenic plants expressing swapped fluorescent markers: *IDH-RFP* and *GFP-ATG8a*. We observed a similar increase in IDH-RFP-associated autophagic bodies after UVB damage (Supplementary Fig. S5). Overall, our monitoring of autophagosomes and mitochondrial fluorescent markers supports the notion that mitochondria are massively transported into the vacuole as autophagosome cargo in UVB-damaged leaves.

### Depolarized mitochondria persist in the cytoplasm of UVB-exposed *atg* leaves

We then used a membrane potential (ΔΨm)-dependent mitochondrial dye, tetramethylrhodamine ethyl ester (TMRE), to assess the quality of mitochondria in UVB-damaged plants. TMRE is positively charged and thus accumulates in active mitochondria, which have a relative negative charge (Scaduto and Grotyohann 1999). Depolarized or inactive mitochondria, however, have decreased membrane potential and fail to accumulate TMRE.

We stained leaves from *Pro35S:MT-GFP* transgenic plants with TMRE (Fig.�7). In untreated control leaves, TMRE stained almost all mitochondria, irrespective of genotype (Fig.�7A). In WT plants, most mitochondria showed TMRE signals even 1 d after UVB exposure (Fig.�7A). By contrast, in *atg* plants, a subset of mitochondria showed no TMRE signals after UVB exposure (Fig.�7A). We counted the mitochondria stained by MT-GFP and by TMRE (Fig.�7B) and used these numbers to calculate the proportion of TMRE-positive mitochondria among the MT-GFP-tagged mitochondria (Fig.�7C). After UVB damage, this proportion decreased to 84.0 � 2.3% in WT plants and 38.6 � 2.6% and 40.1 � 2.6% in *atg5* and *atg7* plants, respectively (Fig.�7C). Similar results were obtained from a comparison between WT and *atg5* expressing *IDH-GFP* (Supplementary Fig. S6). These findings indicate that UVB-damaged mitochondria were selectively eliminated via autophagy to prevent the cytoplasmic accumulation of dysfunctional, depolarized mitochondria.

**Fig. 7:**
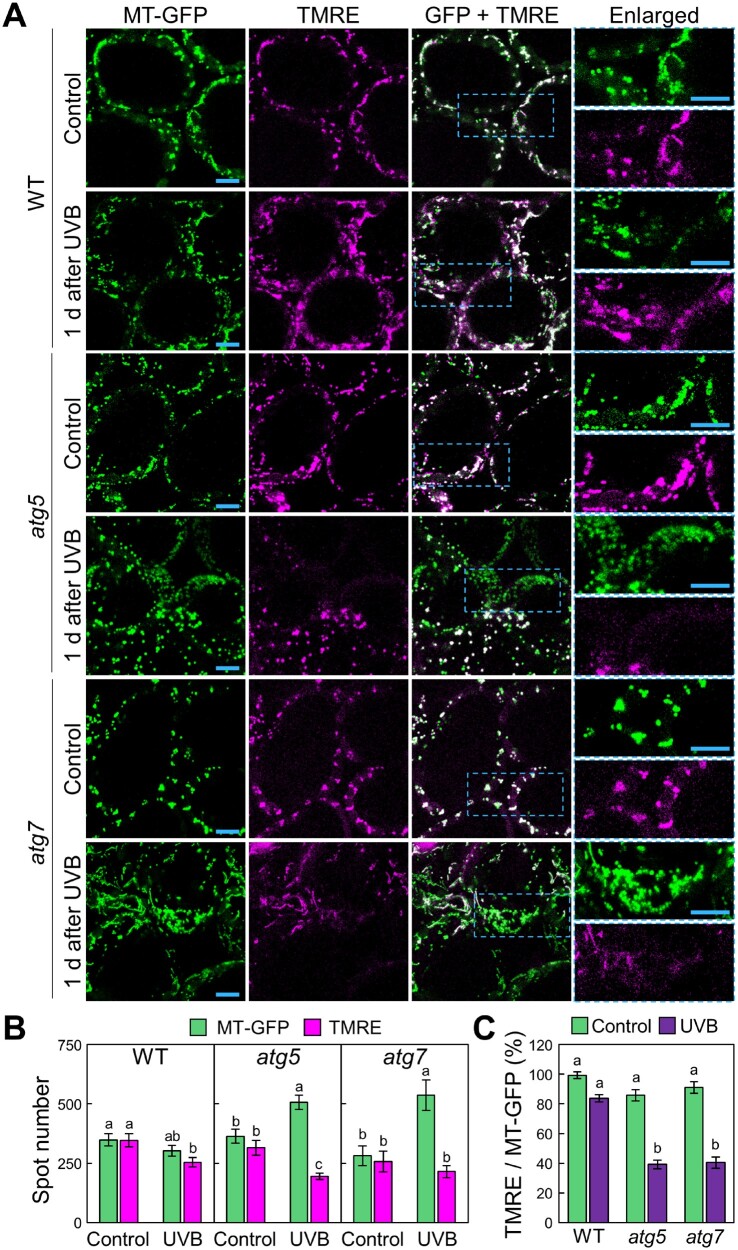
Autophagy reduces the fraction of depolarized mitochondria caused by UVB damage. (A) Confocal images of mesophyll cells expressing *MT-GFP* and stained with TMRE from WT, *atg5* and *atg7* untreated control plants or plants 1 d after a 1-h UVB (1.5 W m^−2^) exposure. Green, mitochondrial GFP; magenta, TMRE. Orthogonal projections created from *z*-stack images are shown. Scale bars = 10 μm. The area indicated by a dashed blue box is expanded to the right of each panel. (B) Number of MT-GFP-labeled or TMRE-labeled mitochondria obtained from the observation described in (A) (� SE, *n* = 4). (C) Proportion of the number of TMRE particles in MT-GFP particles in (B). Different letters in each graph denote significant differences based on Tukey’s test (*P* < 0.05).

### Plant mitophagy contributes to mitochondrial quality control caused by the lack of a mitochondrion-associated protein

UVB directly causes oxidative damage in various intracellular components, including mitochondria, peroxisomes, chloroplasts and the nucleus (Fig.�2; Izumi et al. 2017). Here, we detected the contribution of mitophagy to maintain mitochondrial quality control when more mitochondria-specific defects occur. To this end, we used a mutation in *FRIENDLY*, a member of the CLUSTERED MITOCHONDRIA superfamily, which is essential for the correct distribution and function of mitochondria (Logan et al. 2003, El Zawily et al. 2014).

We first checked the morphology of mitochondria in 18-day-old WT or *friendly* leaves via TEM and detected large mitochondrial clusters specifically in the *friendly* mutant background, and not in WT plants (Fig.�8A). We also detected these structures by imaging the fluorescent mitochondrial marker IDH-GFP (Fig.�8B, arrowheads). We then generated *friendly atg5* and *friendly atg7* double mutant plants expressing IDH-GFP and visualized their leaves under the fluorescence microscopy. Large clusters of mitochondria appeared in *friendly* and *friendly atg* plants without any stress treatment (Fig.�8B), but they were more prevalent in *friendly atg* double mutants relative to the *friendly* single mutant (Fig.�8C), suggesting that autophagy contributes to rescuing the mitochondrial defects caused by the loss of FRIENDLY function, a protein that underlies proper mitochondrial function.

**Fig. 8:**
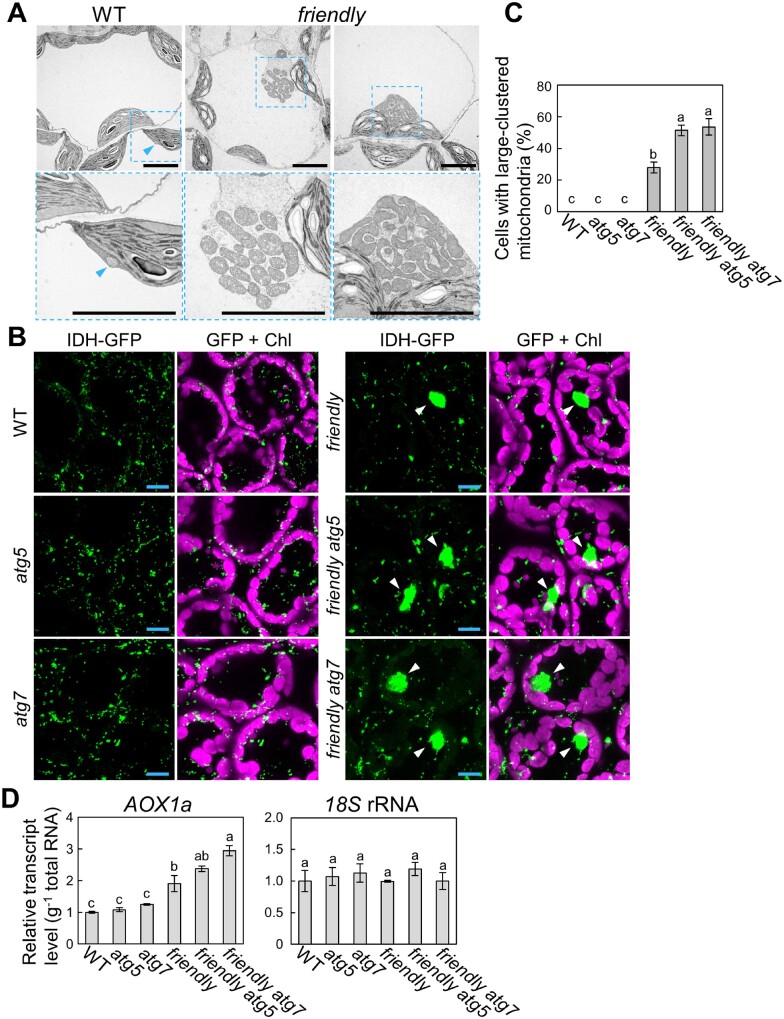
Autophagy alleviates the appearance of clustered mitochondria due to the *friendly* mutation. (A) Ultrastructure of mitochondria in mesophyll cells from WT and *friendly* plants. Ultra-thin sections from third rosette leaves were viewed by TEM. Scale bars = 5 �m. For *friendly* mutants, two representative images are shown. The area indicated by a dashed blue box is expanded below each panel. Blue arrowheads indicate non-clustered mitochondria in the cytoplasm. (B) Confocal images of leaves from 18-day-old *IDH-GFP*-expressing WT, *atg5*, *atg7*, *friendly*, *friendly atg5* and *friendly atg7* plants. Green, IDH-GFP; magenta, chlorophyll autofluorescence (Chl). Scale bars = 10 �m. White arrowheads indicate clustered mitochondria in the cytoplasm. (C) Proportion of cells with clustered mitochondria measured in leaves of 18-day-old untreated plants (� SE, *n* = 4). Different letters denote significant differences based on Tukey’s test (*P* < 0.05). (D) Transcript levels for *AOX1a* in leaves of 18-day-old untreated plants of the various genotypes (� SE, *n* = 3), relative to the values from WT leaves, which were set to 1. The level of *18S* rRNA was measured as an internal control. Different letters in each graph denote significant differences based on Tukey’s test (*P* < 0.05).

Another gene required for nominal mitochondrial function is *ALTERNATIVE OXIDASE 1a* (*AOX1a*), which encodes a non-proton-pumping terminal oxidase in the mitochondrial respiratory chain and is widely considered a stress-responsive gene related to mitochondria (Clifton et al. 2005, Van Aken et al. 2009). We thus evaluated the *AOX1a* transcript level in WT, *atg5*, *atg7*, *friendly*, *friendly atg5* and *friendly atg7* leaves. *AOX1a* transcript abundance rose in *friendly* mutants compared to WT plants and further increased in *friendly atg7* double mutants (Fig.�8D), indicating an increase in mitochondrion-related stress in the double mutants.

We therefore turned to TMRE staining to assess mitochondrial activity in the absence of any stress treatment (Fig.�9A). In WT, *atg5* and *atg7* leaves, IDH-GFP-labeled mitochondria accumulate TMRE signal. By contrast, *friendly* mutants contained some mitochondria without TMRE signal, indicating an increase in heterogeneity in the whole mitochondrial population, resulting from the appearance of functionally altered mitochondria. We further observed that *friendly atg5* and *friendly atg7* mutants contained even higher proportions of such depolarized mitochondria (Fig.�9A). We also evaluated the ratio of the volume of TMRE-labeled mitochondria to that of IDH-GFP-labeled mitochondria for each genotype (Fig.�9B). The proportion of functional mitochondria accumulating TMRE signal was even lower in *friendly atg* double mutants than in *friendly* single mutants. These results support the notion that autophagy participates in the elimination of mitochondria whose function has become compromised by the loss of mitochondrial quality control in Arabidopsis leaves.

**Fig. 9:**
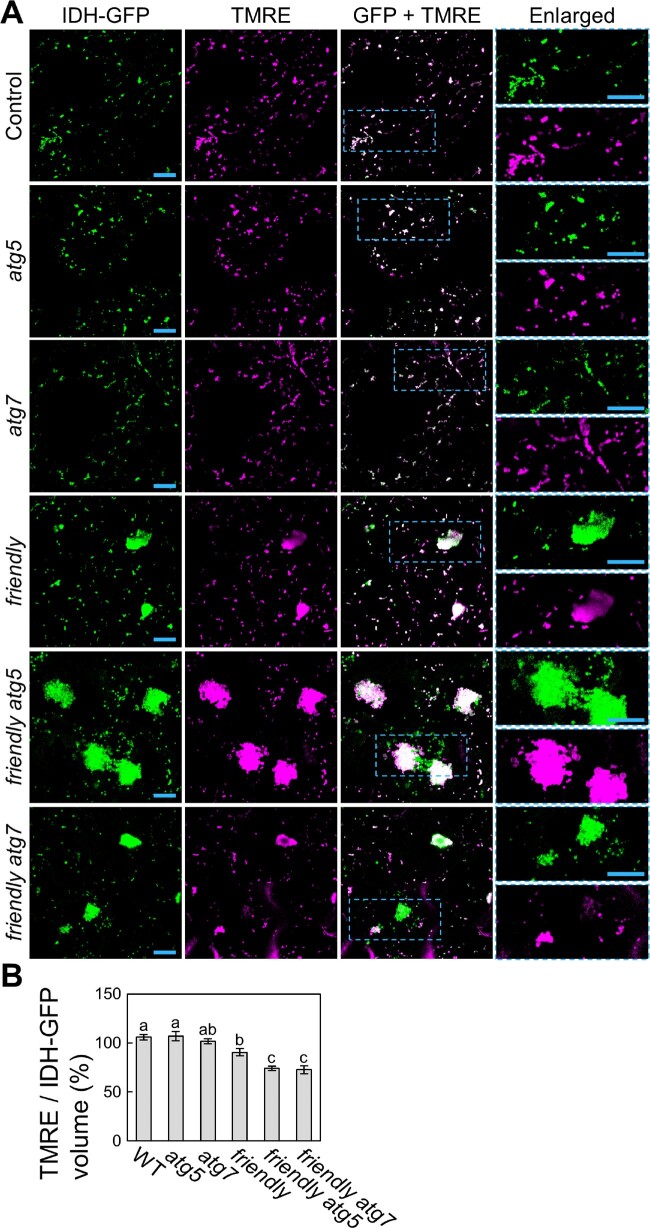
Autophagy alleviates the accumulation of depolarized mitochondria due to *friendly* mutation. (A) Confocal images of TMRE-stained mesophyll cells from WT, *atg5*, *atg7*, *friendly*, *friendly atg5* and *friendly atg7* plants expressing mitochondrial *IDH-GFP*. Green, IDH-GFP; magenta, TMRE. Orthogonal projections created from *z*-stack images are shown. Scale bars = 10 �m. The area indicated by a dashed blue box is expanded to the right of each panel. (B) Proportion of the total volume from TMRE signals in MT-GFP signals obtained from the observations described in (A) (� SE, *n* = 6). Different letters denote significant differences based on Tukey’s test (*P* < 0.05).

## Discussion

Although we previously showed that autophagy is important for the tolerance of Arabidopsis plants to photooxidative damage caused by UVB exposure (Izumi et al. 2017), the roles of autophagy in the UVB-adaptive response have not been fully explored, as the lack of autophagic elimination of damaged chloroplasts is insufficient to explain the UVB sensitivity of *atg* mutant plants (Fig.�1). In this study, we focused on the relationships between autophagy and the fate of other organelles in Arabidopsis leaves upon exposure to UVB for 1 h. Our analysis led to the finding that dysfunctional mitochondria accumulate in the cytoplasm of autophagy-deficient mutant plants after UVB treatment (Figs.�3–5, 7, Supplementary Fig. S6). We further observed the active transport of mitochondria into the vacuoles of cells in UVB-damaged WT leaves (Fig.�6, Supplementary Fig. S5). Mitochondrion-specific detects caused by the *friendly* mutation, which inactivates a protein required to maintain proper mitochondrial function and distribution, was aggravated by additional loss of autophagy (Figs.�8, 9). These results indicate the participation of mitophagy in mitochondrial quality control in Arabidopsis leaves.

### Autophagic removal of damaged organelles in important in plant tolerance to UVB damage

Previous studies described the importance of pexophagy in ensuring the quality control of leaf peroxisomes, especially during senescence (Kim et al. 2013, Shibata et al. 2013, Yoshimoto et al. 2014). Here, we also detected the contribution of pexophagy to the elimination of UVB-damaged peroxisomes (Fig.�2). We further established that mitophagy prevents the accumulation of depolarized mitochondria. UVB damage also induced chlorophagy (Fig.�1). These observations indicate that autophagy is important in the quality control of multiple organelles in UVB-damaged leaves, as the inactivation of these systems in core *ATG* mutants leads to their susceptibility to UVB damage. Although we did not detect a significant difference in nuclear volume between WT and *atg5* plants (Fig.�2A, B), the involvement of plant autophagy in the degradation of nuclei should be further addressed under various conditions since autophagy targets nuclear components in budding yeast (Mochida et al. 2015). By contrast, HL damage is more specific to chloroplasts since chloroplasts absorb visible light and produce ROS. In fact, GFP-labeled mitochondria did not exhibit any clear changes after HL treatment, although HL induces active chlorophagy (Fig.�1, Supplementary Fig. S3). The different roles played by autophagy in response to UVB or HL damage may explain the different sensitivities of *atg* mutants to UVB or HL stress.

The changes in mitochondrial behavior occurred 1 d after a 1-h exposure to UVB (Fig.�3). At this time point, chlorophagy has not started (Fig.�1). Even over 3 d after UVB treatment for 1 h, few chloroplasts were degraded via chlorophagy (Fig.�1). This difference in the timing of chlorophagy and mitophagy implies that mitochondria may be more sensitive to UVB damage than chloroplasts. Thus, the quick removal of damaged mitochondria by mitophagy might be more important than removal of damaged chloroplasts by chlorophagy during adaptation to UVB-induced damage in Arabidopsis plants.

Why mitochondria are more sensitive to UVB damage compared with chloroplasts remains unclear. Both organelles have their own mechanisms to protect against UVB and the related oxidative stress, such as DNA repair mechanisms and ROS-scavenging systems (Boesch et al. 2011); the differences in such inter-organellar systems might be linked to the observed differences in UVB sensitivity. However, mitochondrial and chloroplast DNA repair systems are not fully understood (Boesch et al. 2011). In budding yeast, the loss of mitophagy causes an increase in mitochondrial DNA mutations (Kurihara et al. 2012). Given that UVB causes DNA damage, and the loss of DNA repair enzymes leads to susceptibility to UVB stress in plants (Supplementary Fig. S1; Landry et al. 1997, Willing et al. 2016), plant mitophagy might be especially important in the elimination of damaged mitochondrial DNA.

### UVB damage triggers mitophagy in leaves

The mitochondrial population changed after UVB damage but did not change after damage incurred by HL (Fig.�3, Supplementary Fig. S3), suggesting that UVB-related damage in particular causes a specific signal that activates mitophagy. UVB damages various types of proteins, lipids and DNA directly (Takahashi and Badger 2011, Kataria et al. 2014). Such direct damage to macromolecules might be linked to the initiation of mitophagy. In human skin cells, mitochondria are the major site of damage by UVB exposure; in keratinocytes, a type of human skin cell, UVB-induced inactivation of manganese-containing superoxide dismutase, an ROS-scavenging enzyme, activates mitophagy to prevent the accumulation of damaged mitochondria (Dhar et al. 2019). Thus, the accumulation of ROS that are associated with the dysfunction of mitochondrial ROS-scavenging enzymes might be similarly involved in the induction of mitophagy in plant mesophyll cells. The relationships among UVB damage, ROS production and plant mitophagy remain to be further examined.

We observed the accumulation of small, fragmented mitochondria in UVB-damaged *atg* plant leaves (Fig.�4), suggesting that, in WT leaves, mitophagy quickly eliminates the fragmented mitochondria caused by UVB damage. A previous study using human keratinocytes observed increased fragmentation of mitochondria in response to UVB damage, mediated by dynamin-related protein1, an essential GTPase for mitochondrial fission (Juge et al. 2016), orthologs of which are found in Arabidopsis (Arimura 2018). The relationship between mitophagy and the mitochondrial fission machinery in plants will be an interesting topic for further research to understand the triggers of plant mitophagy.

### Mechanism for mitophagy

Relative to yeast and mammalian cells, in which mitophagy is well established and selectively degrades mitochondria (Yamano et al. 2016, Pickles et al. 2018), the understanding of selective mitophagy in plant cells is still rudimentary. The current study shows that plants have a selective mitophagy pathway for the removal of dysfunctional mitochondria. We hope in the future to investigate the recognition mechanism responsible for the removal of damaged mitochondria in plants. In yeast cells, ATG32 acts as an autophagic receptor bridging mitochondria and the autophagosomal membrane to facilitate mitophagy (Kanki et al. 2009, Okamoto et al. 2009). Similarly, specific mitophagy receptors exist in mammalian cells. During hypoxia, a mitochondrial outer-envelope protein, FUN14 domain-containing protein 1, acts as an ATG32 counterpart, as its dephosphorylation induces mitophagy (Liu et al. 2012). Outer-envelope proteins that are classified as mammalian B-cell lymphoma 2 (BCL2) family members, BCL2/adenovirus E1B 19 kDa-interacting protein 3 (BNIP3), BNIP3L/Nix and Bcl2-like 13 (Bcl2L13) also bridge mitochondria and mammalian ATG8 homologs to induce mitophagy (Schweers et al. 2007, Sandoval et al. 2008, Novak et al. 2010, Murakawa et al. 2015). FK506 binding protein 8 (FKBP8) transiently connects dysfunctional mitochondria that are depolarized in response to treatment with uncoupler drugs and autophagosomal membranes for their removal (Bhujabal et al. 2017). Another mitophagy route that has been extensively studied is PTEN-induced kinase 1 (PINK1)/Parkin-mediated mitophagy, during which the mitochondrion-targeted kinase PINK1 accumulates on depolarized mitochondria and stimulates the ubiquitination of mitochondrial surfaces by the cytosolic ubiquitin ligase Parkin (Pickles et al. 2018). Ubiquitinated mitochondria are then targeted to be engulfed by autophagosomes for degradation. To date, however, no obvious plant homologs for such players in mitophagy have been characterized.

A recent preprint article reported the contribution of mitophagy to the quality control of mitochondria in Arabidopsis roots treated with an uncoupler (Ma et al. 2020). Uncouplers act as proton ionophores; they dissipate the proton gradient across the mitochondrial inner membrane, resulting in the loss of ATP synthesis capacity within mitochondria. The results from that study support the main conclusion of the present study (i.e. mitophagy occurs in plant cells to reduce the numbers of damaged mitochondria). Here, we observed an additive effect on the morphology and function of mitochondria in *friendly atg* double mutant leaves (Figs.�8, 9), suggesting the independent functions of FRIENDLY and autophagy in leaves. By contrast, the study in uncoupler-treated roots indicated that FRIENDLY directly contributes to the induction of mitophagy (Ma et al. 2020). Therefore, the different roles of FRIENDLY in the induction of mitophagy in distinct stresses or organs should be further elucidated. As in mammals, several types of regulators and receptors might be involved in the induction of plant mitophagy in different tissues, growth stages and stress conditions.

### Tools for the study of plant mitochondrial quality control

UV radiation causes direct damage to various types of intracellular macromolecules (Takahashi and Badger 2011, Kataria et al. 2014). Such damage is involved in the rapid acceleration of cell death, and this phenomenon is further enhanced in *atg* mutant plants (Fig.�1, Supplementary Fig. S1). This close association between UVB damage and cell death complicates further analysis of the mechanism of mitophagy. For instance, autophagy provides a specific route from the cytoplasm to the vacuole for the degradation of autophagosomal cargos; however, cell death subsequently causes mixing of cytoplasmic components and vacuolar lytic activity, even though autophagy is not activated. Thus, the phenomena resulting from cell death hinder the biochemical evaluation of autophagy flux, such as immunoblot detection of the degradation of organellar proteins or fluorescent protein-labeled autophagic cargos. Therefore, in the current study, we mainly evaluated mitochondrial populations and quality in living cells of Arabidopsis rosette leaves through microscopy. To advance the elucidation of the plant mitophagy mechanism, a more stable assay system may be useful, such as the *friendly* mutant background used in this study (Figs.�8, 9). Previous research has demonstrated that these mutants have impaired mitochondrial function and morphology, along with altered expression of autophagy-related genes (El Zawily et al. 2014). Consistent with these results, the current study indicates that autophagy plays a supportive role in mitochondrial quality control in *friendly* mutants (Figs.�8, 9). The *friendly* mutation causes an increase in heterogeneity in the mitochondrial population as a result of the appearance of functionally altered mitochondria (El Zawily et al. 2014). We also observed such heterogeneity: a subset of both clustered mitochondria and non-clustered mitochondria failed to accumulate TMRE signals in *friendly* leaves (Fig.�9A). The additional loss of autophagy due to *atg5* or *atg7* mutations aggravated this mitochondrial heterogeneity (Fig.�9A, B), indicating the importance of mitophagy to reduce the numbers of dysfunctional mitochondria. Thus, our data suggest that plant lines carrying mutations related to mitochondrial quality may provide an efficient experimental system for the study of plant mitophagy.

In summary, the current study establishes that plant autophagy plays a role in the quality control of leaf mitochondria. As plants take in sunlight for photosynthesis, leaf mitochondria are constantly exposed to UVB damage. Thus, autophagy helps maintain mitochondrial function during plant growth by removing damaged mitochondria. Further focus on the precise mechanisms underlying this mitophagic process will help elucidate plant adaptation strategies in the face of natural sunlight-induced damage.

## Materials and Methods

### Plant materials

Plants were grown on soil in chambers at 23�C under a 12-h light/12-h dark photoperiod with fluorescent lamps or LEDs (120 μmol m^−2^ s^−1^). The Arabidopsis T-DNA insertion lines for *atg5* (*atg5-1*; SAIL_129_B07), *atg7* (*atg7-2*; GABI_655B06), *atg2* (*atg2-1*; SALK_076727), *atg10* (*atg10-1*; SALK_084434), *atg12ab* (*atg12a*; SAIL_1287_A08, *atg12b*; SALK_003192), *atg18a* (GABI_651D08) and *friendly* (SALK_046271) were previously described (Logan et al. 2003, Thompson et al. 2005, Xiong et al. 2005, Inoue et al. 2006, Phillips et al. 2008, Hofius et al. 2009, Chung et al. 2010). The T-DNA insertion line of *phr* (*phr-3*; WiscDsLox368H08) was obtained from the Arabidopsis Biological Resource Center and characterized in this study (Supplementary Fig. S1). Transgenic Arabidopsis expressing ankyrin-like protein (At4g19150)-GFP driven by the CaMV 35S promoter (*Pro35S:GFP-At4g19150*), *IDH-GFP* driven by the *IDH* promoter (*ProIDH:IDH-GFP*), MT-GFP driven by CaMV *35S* promoter (*Pro35S:MT-GFP*), *MFP2* (At3g06860)-GFP driven by the CaMV *35S* promoter (*Pro35S:GFP-MFP2*) and *VHP1-mGFP* driven by the *VHP1* promoter (*ProVHP1:VHP1-mGFP*) were previously described (Kohler et al. 1997, Cutler et al. 2000, Segami et al. 2014, Hirota et al. 2018). All other plant lines expressing organellar fluorescent markers were generated as follows.

For plants expressing CaMV35S promoter-driven *SWIB2-GFP* (*Pro35S:SWIB2-GFP*), the coding region of *SWIB2* (At2g14880) was amplified from Arabidopsis cDNA by PCR using the primers SWIB2-F and SWIB2-R (Supplementary Table S1). The amplicon was cloned into pENTR/D/TOPO (Invitrogen) and transferred to the vector pGWB505 (Nakagawa et al. 2007), in an LR recombination reaction.

For the *IDH* promoter-driven mitochondrial *IDH-RFP* construct, a DNA fragment comprising 396-bp upstream from the start codon to the region just before the stop codon of *IDH1* (At4g35260) was amplified from Arabidopsis genomic DNA by PCR using the primers IDH1-F and IDH1-R (Supplementary Table S1), cloned into pENTR/D/TOPO and transferred to the vector pGWB553 (Nakagawa et al. 2007).

For *UBQ10* promoter-driven *GFP-ATG8a* or *RFP-ATG8a*, the coding sequence of *ATG8a* (At4g21980.1) was amplified from Arabidopsis cDNA by PCR using the primers attB1ATG8a-F and attB2ATG8a-R (Supplementary Table S1) and then cloned into the vector pDONR221 (Invitrogen) in a BP clonase reaction (Invitrogen) according to the manufacturer’s instructions, or transferred to the vector pUBN-GFP-Dest or pUBN-RFP-Dest (Grefen et al. 2010), respectively.

The resulting vectors were introduced into Agrobacterium (*Agrobacterium tumefaciens*) strain GV3101 and then introduced into Arabidopsis ecotype Columbia (Col-0) by the floral-dip method (Clough and Bent 1998). Transgenic plants expressing two types of fluorescent markers were generated by sexual crossing or additional transformations.

### Light treatment

UVB exposure was provided with UVB-fluorescent tubes (FL20SE; Toshiba or GL20SE; Sankyo Denki). Strong light exposure was provided by a xenon light source (MAX-303; Asahi Spectra) equipped with a mirror module (MAX-VIS; Asahi Spectra) to extract visible light (wavelength 385–740 nm) and a rod lens (RLQL80-1; Asahi Spectra) to emit light with uniform intensity in a chamber. The intensity of UVB or visible light was measured with a data logger (LI-1400; Li-Cor) equipped with a UVB sensor (SD204B; Li-Cor) or a photosynthetic photon flux density sensor (LI-190SA; Li-Cor), respectively. After the treatment, plants were cultivated in the indicated growth conditions until the analysis.

For ConA treatment to suppress lytic activity in the vacuole, MES-NaOH (pH 5.5) containing 1 �M ConA was infiltrated immediately after UVB treatment into leaves with a 1-ml syringe and the leaves incubated under the indicated growth conditions. After 1 or 2 d, leaf mesophyll cells were observed under the confocal microscopy.

### RT-qPCR

Evaluation of *PHR*, *GFP*, *IDH1* and *ATG5* mRNA and *18S* rRNA levels by RT-qPCR (Supplementary Fig. S1, Fig.�3) was performed as described previously (Izumi et al. 2017). Total RNA was isolated from rosette leaves using the RNeasy kit (Qiagen) and then subjected to first-strand cDNA synthesis using random hexamer and oligo(dT) primers with the PrimeScript RT Reagent Kit with gDNA Eraser (Takara). An aliquot of the synthesized cDNA derived from 4.0 ng total RNA was subjected to RT-qPCR analysis with the KAPA SYBR FAST qPCR Kit (KAPA Biosystems) using a real-time PCR detection system (CFX96; Bio-Rad). The level of *18S* rRNA was used as an internal control (Izumi et al. 2012). Gene-specific primers were as in previous studies (Thirkettle-Watts et al. 2003, Clausen et al. 2004, Lemaitre and Hodges 2006, Rose et al. 2006, Kwon et al. 2010, Izumi et al. 2012, Izumi et al. 2013, Supplementary Table S1).

The evaluation of *AOX1a* mRNA and *18S* rRNA levels was performed as follows. Total RNA was isolated from rosette leaves using the Maxwell RSC Plant RNA Kit (Promega) and subjected to first-strand cDNA synthesis using random hexamer and oligo(dT) primers with the PrimeScript RT Reagent Kit (Takara). An aliquot of the synthesized cDNA derived from 10 ng total RNA was subjected to RT-qPCR analysis with the SsoAdvanced Universal SYBR Green Supermix (Bio-Rad) using a real-time PCR detection system (CFX96; Bio-Rad). The level of *18S* rRNA was measured as an internal control (Izumi et al. 2012). The primer sequences for RT-qPCR analysis are listed in Supplementary Table S1.

### Measurement of PSII quantum yield by *F*_v_*/F*_m_

The maximum quantum yield of PSII (*F_v_/F_m_*) was calculated using a pulse-modulated fluorometer (Junior-PAM; Walz) at room temperature. First, the plants were incubated in the dark for >30 min, and then *F*_0_ and subsequently *F*_m_ in third rosette leaves were measured by saturating pulse quenching analysis with evenly pulsed measuring light and a saturating pulse emitted from the fluorometer through plastic fiber.

### Image analysis with laser scanning confocal microscopy

Laser scanning confocal microscopy (LSCM) was performed with a Carl Zeiss LSM800 system equipped with a C-apochromat LD63� water-immersion objective lens [numerical aperture (NA) = 1.15; Carl Zeiss] or a Nikon C2 system equipped with a CFI Apochromat LWD Lambda S 40�C WI (NA = 1.15; Nikon). For UVB treatment, 18-day-old plants were subjected to UVB exposure and the third rosette leaves were visualized under the microscopy. For strong visible light treatment, 18-d-old plants (Fig. 1) or 14-day-old plants (Supplementary Fig. S3) were exposed to strong light and the third or second rosette leaves were observed, respectively.

For the quantitative evaluations of organelle size or population, three-dimensional images for nuclei (101.4 �m � 101.4 �m � 30 �m), plastid nucleoids (35 �m � 35 �m � 15 �m), peroxisomes (163.8 �m � 163.8 �m � 15 �m) and mitochondria (101.4 �m � 101.4 �m � 15 �m) were obtained by *z*-stacks. Four individual plants were observed and four images were obtained from different parts of a single leaf for each plant. For evaluation of the appearance of mitochondrial clusters caused by the *friendly* mutation, four regions (163.8 �m � 163.8 �m � 15��m) per plant were monitored to count the cells with large-clustered mitochondria (indicated by white arrowheads in Fig.�8B). The quantification for three-dimensional images was performed with the Imaris software (Bitplane). For plastid nucleoids, the strong signal derived from plastids in epidermal cells was eliminated from the quantification, and the nucleoids associated with chloroplasts in mesophyll cells were counted. 2D orthogonal projections were produced in the Zeiss LSM ZEN blue browser software or Nikon NIS elements software.

For the quantitative evaluations of autophagic structures visualized with GFP- or RFP-ATG8, four different regions (110.4 �m � 50.7 �m each) per plant were monitored with LSCM by adjusting the focus to count the number of puncta.

### Image analysis with two-photon excitation laser-scanning microscopy with a spinning-disk scanner unit

Image analysis with two-photon excitation confocal microscopy with spinning disk unit was performed as previously described (Otomo et al. 2015, Sasaki et al. 2019). The GFP and chlorophyll signals were excited by 920-nm femtosecond light pulses generated by a mode-locked titanium-sapphire laser light source (Mai Tai eHP DeepSee; Spectra Physics). The fluorescent signals were observed under an inverted microscope (Ti-E; Nikon) equipped with a spinning-disk scanner with 100-μm-wide pinholes aligned on a Nipkow disk (CSU-MPϕ100; Yokogawa Electric) and an objective lens (CFI Plan Apo IR 60XWI 60�, NA aperture = 1.27; Nikon). Then, images of GFP and chlorophyll signals were simultaneously obtained using image-splitting optics (W-View Gemini; Hamamatsu Photonics), including a dichroic mirror (FF580-FDi01-25 � 36; Semrock) and bandpass filters (BrightLine 528/38; Semrock and BrightLine 685/40; Semrock), and an EM-CCD camera (iXon Ultra 897; Andor Technology). *z*-Scans were performed with a piezo actuator (Nano-Z100 NIK-S2191; Mad City Labs). For the quantitative evaluation of mitochondrial size or population, three-dimensional images (90 �m � 55 �m � 25 �m) were obtained from *z*-stacks. The acquired images were analyzed using NIS-Elements C software (Nikon) or Imaris software (Bitplane).

### TEM

TEM was performed as previously described (Izumi et al. 2017). Briefly, leaves were fixed with 50 mM cacodylate buffer (pH 7.4) containing 2% glutaraldehyde and 2% paraformaldehyde overnight at 4�C. The samples were rinsed with 50 mM cacodylate buffer and post-fixed with 2% osmium tetroxide for 3 h at 4�C. Following dehydration in ethanol gradient solutions (50%, 70%, 90% and 100%), samples were embedded and polymerized in resin at 60�C for 2 d. Ultra-thin sections (70 or 80 nm) were produced from the resin blocks with a diamond knife attached to an ultramicrotome (Ultracut UCT; Leica). These sections were placed on copper grids and stained with 2% uranyl acetate, followed by the observations under a transmission electron microscope (JEM-1400Plus; JEOL) equipped with a CCD camera (VELETA; Olympus). For the quantitative evaluations of mitochondrial size or population, 12 images of fixed area (14.75 �m � 11 �m) were analyzed.

### TMRE staining

To monitor the ΔΨm in leaf mesophyll cells, third rosette leaves were infiltrated 200 nM TMRE using a 1-ml syringe. Leaves were washed two to three times with distilled water and subjected to the observation under the confocal microscopy.

### Statistical analysis

Statistical analysis in this study was performed with the software JMP (SAS Institute). Student’s *t-*test was used for the comparison of paired samples, while Tukey’s test or Dunnett’s test was used for the comparison of multiple samples, as indicated in figure legends.

## Supplementary Data

Supplementary data are available at PCP online.

## Funding

Japan Society for the Promotion of Science (JSPS) KAKENHI (grant numbers JP16H06280, JP17H05050, JP18H04852, JP19H04712, JP20H04916 and JP20K21322 to M.I., JP19J01681, JP20K15501 and JP20H05352 to S.N., JP20H05306 to H.I. and JP17H06350 to S.H.); the JSPS Research Fellowship for Young Scientists (to S.N.); Japan Science and Technology Agency (JST) PRESTO (grant number JPMJPR16Q1 to M.I.); the Cooperative Research Program of ‘NJRC Mater. & Dev.’ (to M.I.); and the Joint Research by Exploratory Research Center on Life and Living Systems (ExCELLS Program Number 20-314 to M.I.).

## Supplementary Material

pcaa162_Supplementary_Data
