# Supplementary material for: Autophagy Contributes to the Quality Control of Leaf Mitochondria
Source: Plant Cell Physiol. 2020 Dec 23;62(2):229–47. doi: 10.1093/pcp/pcaa162 (PMC8112837; doi:10.1093/pcp/pcaa162)
Supplement: pcaa162_Supplementary_Data [file pcaa162_supplementary_data.pdf]

Fig. S1

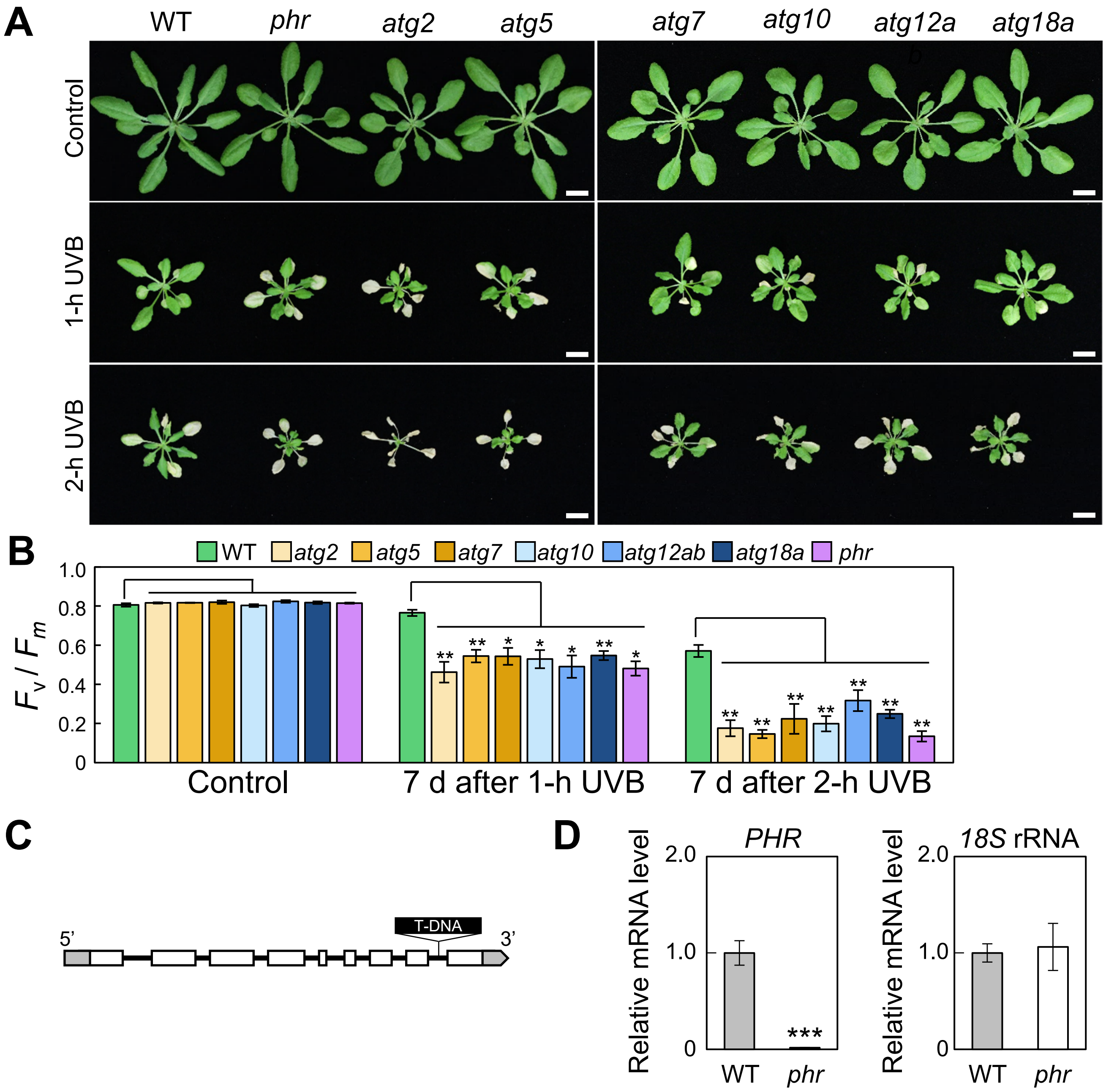

Supplementary Figure S1. Six T-DNA insertion mutants of core *ATG* genes are sensitive to UVB.
(A) Visual phenotypes of Arabidopsis plants 7 d before (control), or after 1-h UVB, or 2-h UVB exposure. Wild-type (WT), *phr*, *atg2*, *atg5*, *atg7*, *atg10*, *atg12a atg12b*, and *atg18a* plants were subjected to UVB exposure (wavelength 280–315 nm) of 1.5 W m^{-2} for 1 or 2 h. Scale bars = 10 mm.
(B) Mean F_v/F_m ratios in the leaves of WT, *atg2*, *atg5*, *atg7*, *atg10*, *atg12a atg12b*, *atg18a,* and *phr* plants 7 d after 0-h (control), 1-h or 2-h UVB treatment (±SE, $n = 4$). Plant photographs in the same treatment groups are shown in Supplemental Figure S1A. Asterisks denote significant differences compared to WT data based on Dunnett's test (*$P < 0.05$, **$P < 0.01$).
(C) Isolation of *PHR*-knockout Arabidopsis plants. The T-DNA insertion site in *phr-3* mutant, in the accession Columbia-0 (WiscDsLox368H08). Gray boxes, open boxes, and lines indicate untranslated regions, exons, and introns, respectively.
(D) Transcript levels of *PHR* and *18S* rRNA in leaves of WT and *phr-3* mutant plants (±SE, $n = 4$). Asterisks denote significant differences based on Student's *t*-test (***$P < 0.001$).

Fig. S2

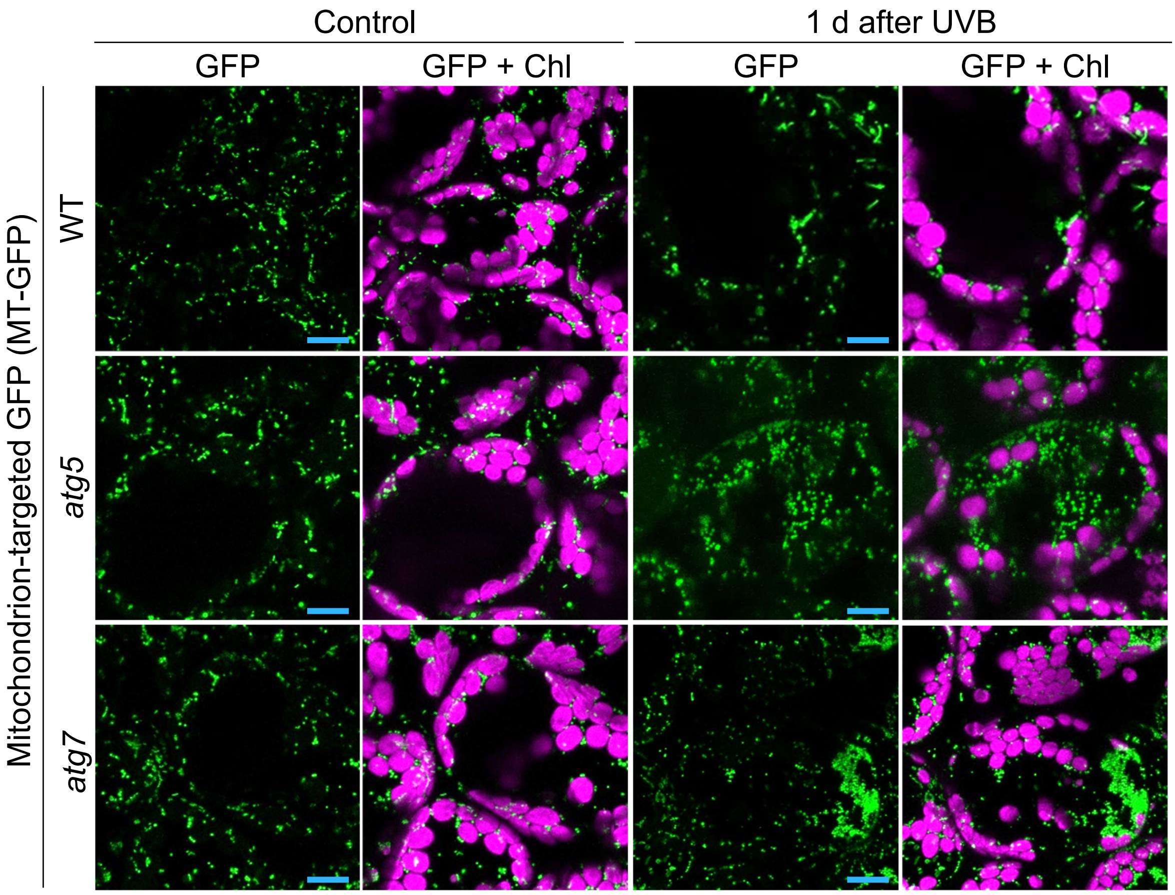

Supplementary Figure S2. The mitochondrial marker expressed from the strong 35S promoter *Pro35S:MT-GFP* also shows elevated mitochondrial population in UVB-damaged *atg* leaves.
Confocal images of mesophyll cells from WT, *atg5*, and *atg7* plants expressing mitochondrion-targeted GFP (*MT-GFP*), either untreated (control) or 1 d after a 1-h UVB (1.5 W m^{-2}) exposure. Green, GFP; magenta, chlorophyll autofluorescence (Chl). Orthogonal projections created from *z*-stack images are shown. Scale bars = 10 µm.

Fig. S3

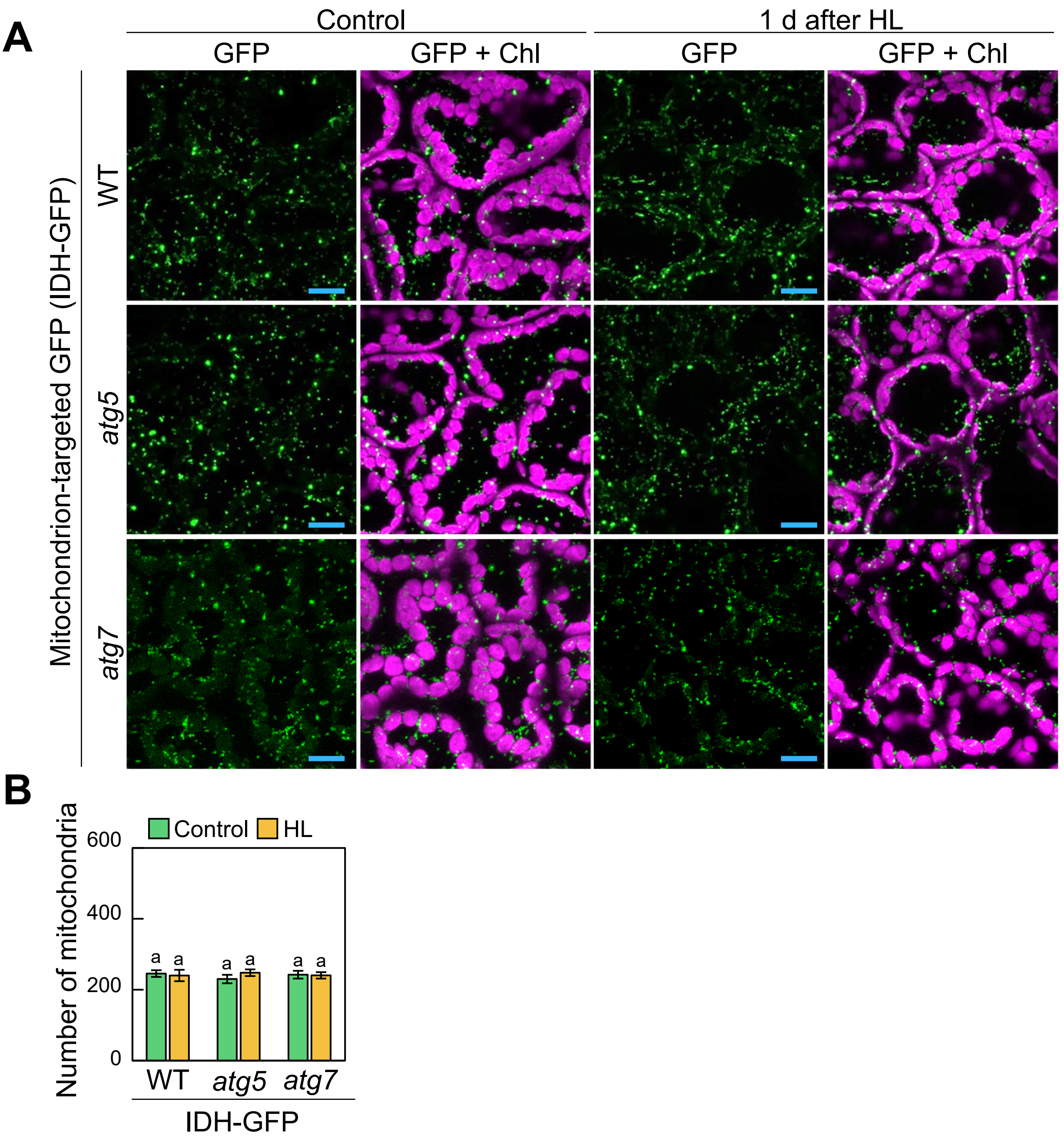

Supplementary Figure S3. Damage from strong visible light does not affect the mitochondrial population in leaves.
(A) Confocal images of mesophyll cells from WT, *atg5*, and *atg7* plants expressing isocitrate dehydrogenase-GFP (*IDH-GFP*), either untreated (control) or 1 d after 2 h HL (2000 μmol m^{-2} s^{-1}) exposure. Green, GFP; magenta, chlorophyll autofluorescence (Chl). Orthogonal projections created from *z*-stack images are shown. Scale bars = 10 μm.
(B) Number of mitochondria obtained from the three-dimensional images described in (A) (±SE, n = 4). Different letters denote significant differences based on Tukey's test ($P < 0.05$).

Fig. S4

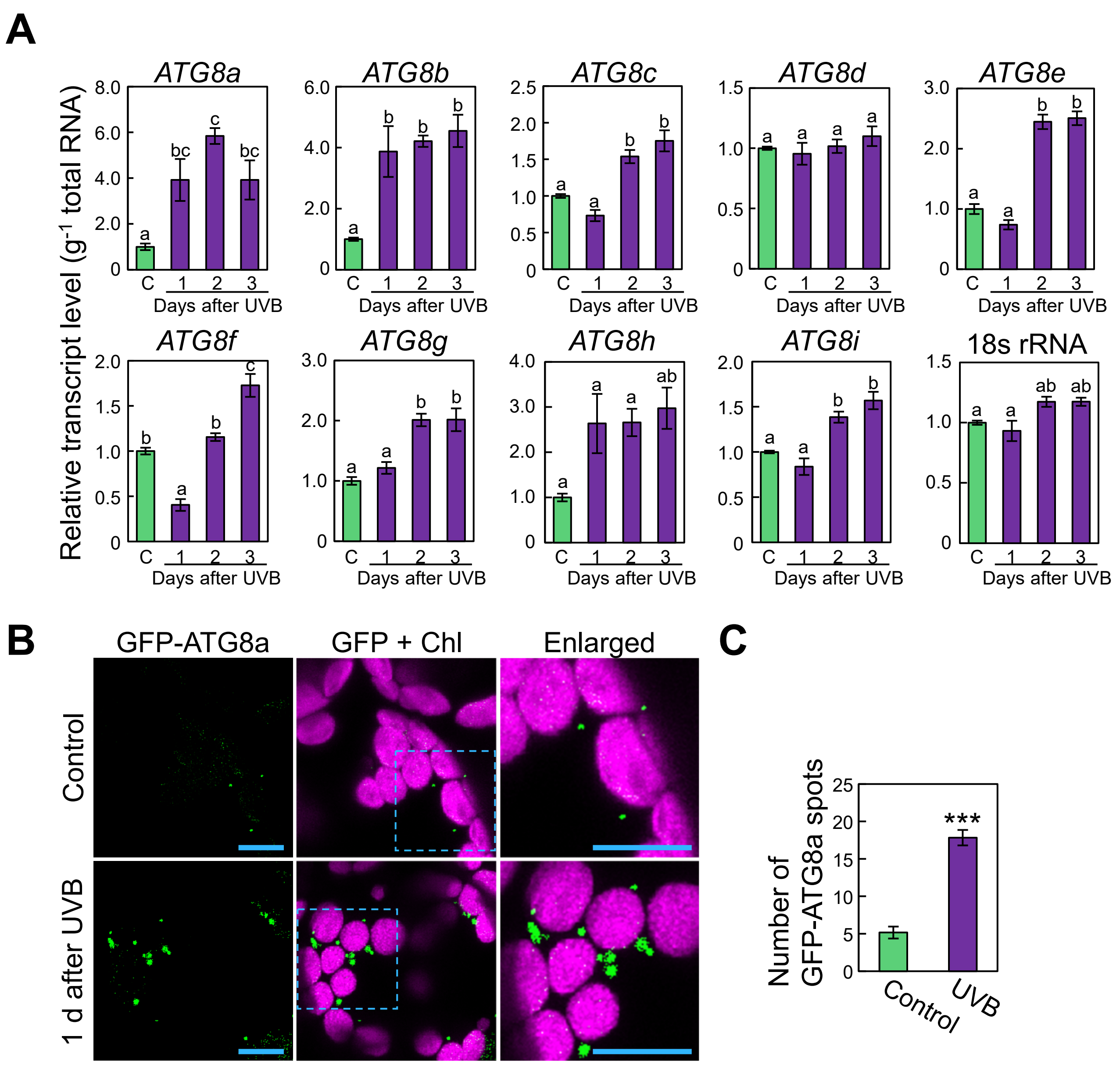

Supplementary Figure S4. UVB damage activates autophagosome formation.

(A) *ATG8* transcript levels measured in leaves from untreated plants (control) or plants 1, 2 or 3 d after 2 h UVB (1.5 W m^{-2}) exposure (±SE, *n* = 3). Transcript levels of the respective genes are shown relative to the values from WT control leaves, which are set to 1. The level of *18S* rRNA was measured as an internal control. Different letters in each graph denote significant differences based on Tukey's test ($P < 0.05$).

(B) Confocal images of mesophyll cells expressing *GFP-ATG8a* from untreated control plants or plants 1 d after a 1-h UVB (1.5 W m^{-2}) exposure. Green, GFP-ATG8a; magenta, chlorophyll autofluorescence (Chl). Scale bars = 10 µm.

(C) Number of GFP-ATG8a-labeled autophagic structures obtained from the observations described in (B) (±SE, *n* = 4). Asterisks denote significant differences between control and UVB-treated plants based on Student's *t*-test ($^{***}P < 0.001$).

Fig. S5

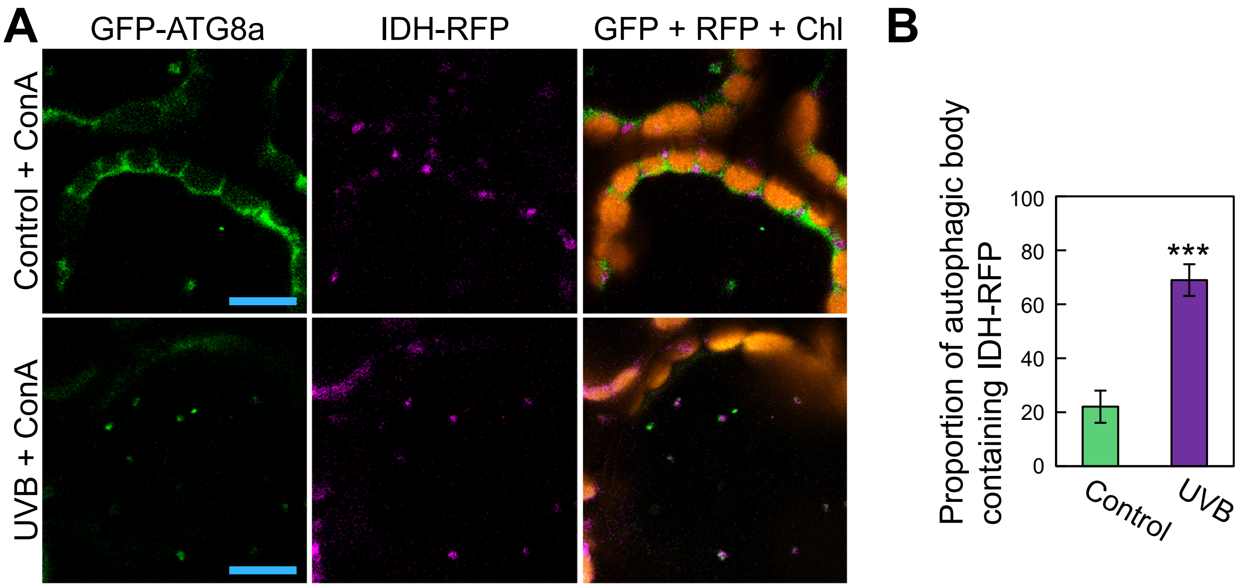

Supplementary Figure S5. Swapped fluorescent markers also show activation of autophagosomal transport of mitochondria in UVB-damaged leaves.

(A) Confocal images of mesophyll cells expressing mitochondrial *IDH-RFP* and autophagosomal *GFP-ATG8a* from concanamycin A (ConA)-treated leaves. Leaves of untreated control plants or plants immediately after a 1-h UVB (1.5 W m^{-2}) exposure were incubated for 2 d with ConA. Green, GFP-ATG8a; magenta, IDH-RFP; orange, chlorophyll autofluorescence (Chl). Scale bars = 10 µm.

(B) Proportion of autophagic bodies associated with mitochondrial IDH-RFP signals obtained from the observations described in (A) (±SE, n = 4).

Fig. S6

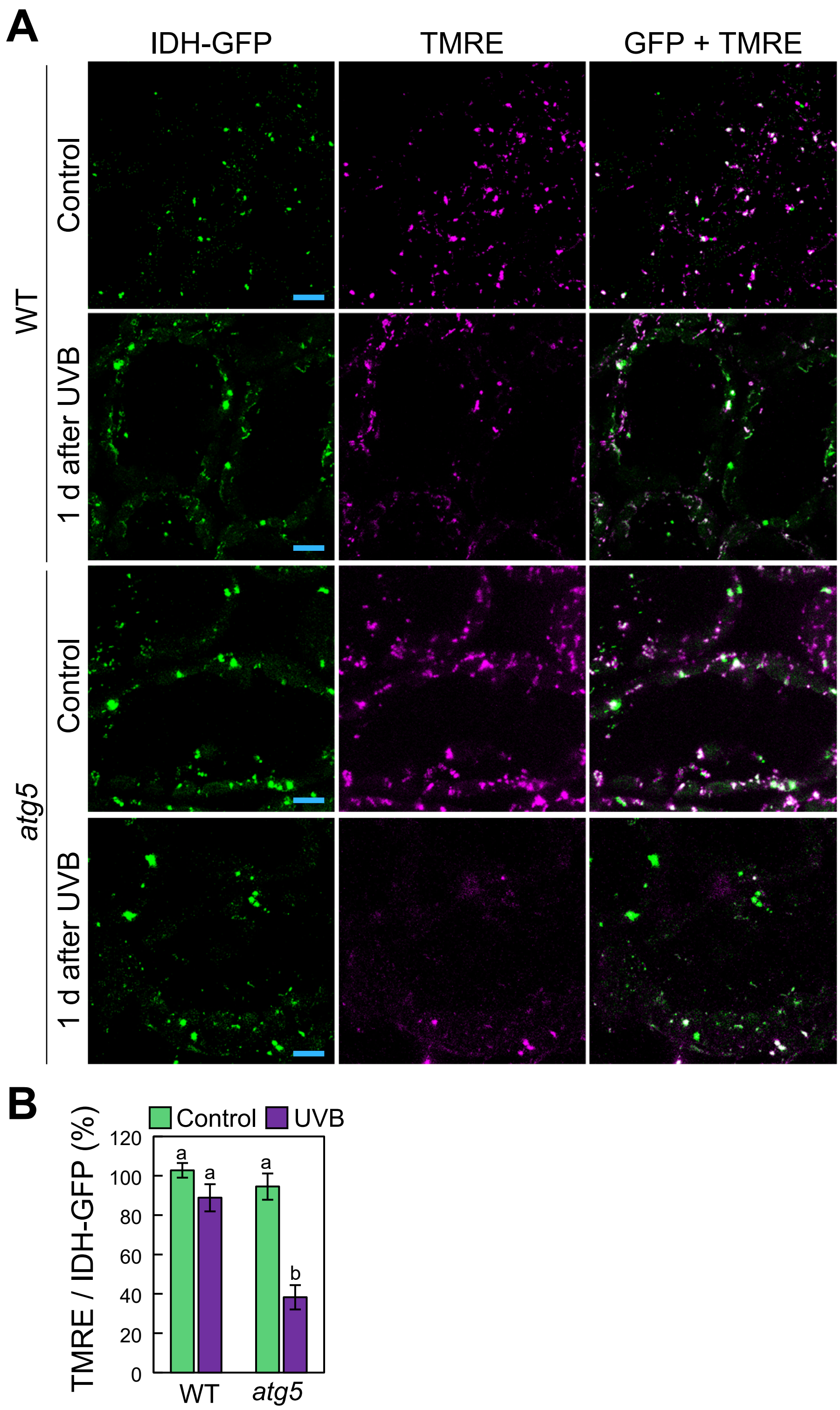

Supplementary Figure S6. The *ProIDH:IDH-GFP* mitochondrial marker indicates the cytoplasmic accumulation of depolarized mitochondria in UVB-damaged *atg* plants.

(A) Confocal images of TMRE-stained mesophyll cells from WT and *atg5* plants expressing *IDH-GFP*, either untreated (control) or 1 d after a 1-h UVB (1.5 W m^{-2}) exposure. Green, mitochondrial IDH-GFP; magenta, TMRE. Orthogonal projections created from *z*-stack images are shown. Scale bars = 10 µm.

(B) Proportion of TMRE particles among the IDH-GFP-labeled particles. Different letters in each graph denote significant differences based on Tukey's test ($P < 0.05$).

Table S1

Gene (Locus)	Primer sequence (5' to 3')		Amplicon size	Reference
	Forward	Reverse	(bp)	
**Gene cloning**				
SWIB2 (At2g14880)	CACCATGGCGGTTTCTTCT	GAGGAAGTGAGGACCGAT	427	1
IDH1 (At4g35260)	CACCTGAAATTCGAGGGTGCAAG	GTCTAGTTTTGCAATGACCGCATC	2136	1
ATG8a (At4g21980.1)	GGGGACAAGTTTGTACAAAAAAGCAGGCTTC ATGGCTAAGAGTTCCTTC	GGGGACCACTTTGTACAAGAAAGCTGGGTTC AAGCAACGGTAAGAGATC	429	1
**qRT-PCR**				
GFP	GTGACCACCTTCACCTACGG	GTCCTTGAAGAAGATGGTGC	126	1
IDH1 (At4g35260)	ATTACGTGTTCCCGCTCTGC	AGGCGCCAACAAACGTAGC	198	2
ATG5 (At5g17290)	ATGGCGAAGGAAGCGGTCA	TCACCTTTGAGGAGCTTTCACAAGG	1014	1
18S rRNA	AATTGTTGGTCTTCAACGAGGAA	AAAGGGCAGGGACGTAGTCAA	74	4
AOX1a (At3g22370)	GACGGTCCGTACGGTTTCG	CTTCTGATTCGCGTCCTCCT	175	5
ATG8a (At4g21980)	CAATTTGTATACGTGGTTCGT	AGCAACGGTAAGAGATCCAA	189	6
ATG8b (At4g04620)	TTGGCCAATTTGTGTACGTT	TCCACCAAATGTGTTCTCTCC	181	6
ATG8c (At1g62040)	TGAGTGCCGAAAAGGCTATC	ACCAAACCAAAGGTGTTCTCT	145	6
ATG8d (At2g05630)	TTTGACTGTTGGCCAGTTTG	AACCCGTCTTCGTCTTTGTG	150	6
ATG8e (At2g45170)	TCTTTAAGATGGACGACGATTTC	CTCAGCCTTTTCCACAATCA	101	7
ATG8f (At4g16520)	TGGGGCAGTTTGTGTATG	GGAACCCATCATCATCCTTTT	144	6
ATG8g (At3g60640)	TGTGATTCGTAAGAGAATCCAAC	CCAAAAGTGTTTTCCCCACT	162	6
ATG8h (At3g06420)	CCAAAGCTCTCTTTGTTTTCG	AAGAACCCGTCTTCTTCCTTG	97	6
ATG8i (At3g15580)	TGTCAACAACACTCTCCCTCA	AACCAAAGGTTTTCTCACTGC	201	6

[1]This study, [2]Lemaitre and Hodges, 2006, [3]Kwon et al., 2010, [4]Izumi et al., 2012, [5]Thirkettle-Watts et al., 2003, [6]Rose et al., 2006, [7]Izumi et al., 2013

Supplemental Table S1. Primer sequences used in this study.
Primer sequences for gene cloning or RT-qPCR analysis.
